# The *Eruca sativa* Genome and Transcriptome: A Targeted Analysis of Sulfur Metabolism and Glucosinolate Biosynthesis Pre and Postharvest

**DOI:** 10.3389/fpls.2020.525102

**Published:** 2020-10-27

**Authors:** Luke Bell, Martin Chadwick, Manik Puranik, Richard Tudor, Lisa Methven, Sue Kennedy, Carol Wagstaff

**Affiliations:** ^1^School of Agriculture, Policy and Development, University of Reading, Reading, United Kingdom; ^2^School of Chemistry Food and Pharmacy, University of Reading, Reading, United Kingdom; ^3^Elsoms Seeds Ltd., Spalding, United Kingdom

**Keywords:** rocket (*Eruca sativa* and *Diplotaxis tenuifolia*), isothiocyanate, postharvest, sulfur assimilation, glucosinolate transport, transcription factor, MYB28, SLIM1

## Abstract

Rocket (*Eruca sativa*) is a source of health-related metabolites called glucosinolates (GSLs) and isothiocyanates (ITCs) but little is known of the genetic and transcriptomic mechanisms responsible for regulating pre and postharvest accumulations. We present the first *de novo* reference genome assembly and annotation, with ontogenic and postharvest transcriptome data relating to sulfur assimilation, transport, and utilization. Diverse gene expression patterns related to sulfur metabolism, GSL biosynthesis, and glutathione biosynthesis are present between inbred lines of rocket. A clear pattern of differential expression determines GSL abundance and the formation of hydrolysis products. One breeding line sustained GSL accumulation and hydrolysis product formation throughout storage. Multiple copies of MYB28, SLIM1, SDI1, and ESM1 have increased and differential expression postharvest, and are associated with GSLs and hydrolysis product formation. Two glucosinolate transporter gene (GTR2) copies were found to be associated with increased GSL accumulations in leaves. Monosaccharides (which are essential for primary metabolism and GSL biosynthesis, and contribute to the taste of rocket) were also quantified in leaves, with glucose concentrations significantly correlated with the expression of numerous GSL-related genes. Significant negative correlations were observed between the expression of glutathione synthetase (GSH) genes and those involved in GSL metabolism. Breeding line “**B**” showed increased GSH gene expression and low GSL content compared to two other lines where the opposite was observed. Co-expression analysis revealed senescence (*SEN1*) and oxidative stress-related (*OXS3*) genes have higher expression in line **B**, suggesting that postharvest deterioration is associated with low GSL concentrations.

## Introduction

Sulfur (S) is a critical macronutrient that plants require for growth and development (Kopriva et al., 2016). Sulfate (SO_4_^2–^) is utilized as a primary means of synthesizing numerous S-containing metabolites, such as amino acids (cysteine and methionine), alkyl-cysteine-sulfoxides, glutathione (GSH), and glucosinolates (GSLs; Frerigmann and Gigolashvili, 2014). GSL compounds are present in species of the order Brassicales, and are abundant in many vegetables and condiments worldwide, such as rapeseed (*Brassica napus*), Chinese cabbage (*Brassica rapa*), cabbage (*Brassica oleracea* var. *capitata*), and broccoli (*B. oleracea* var. *italica*; Yan and Chen, 2007). GSLs are also found in the leafy vegetable *Eruca sativa* (“salad” rocket), which has gained significant popularity amongst consumers over the last 10 years (Bell and Wagstaff, 2014). Rocket is known for its distinctive flavor, aroma, and pungency, and can be eaten raw without the need for cooking (Bell et al., 2017a), which can lead to a loss of nutritional benefits.

Sulfur assimilated by Brassicales plants is thought to be a strong determining factor in the biosynthesis of GSLs (Pandey et al., 2017). GSLs themselves are not bioactive, and are hydrolyzed by myrosinase enzymes (β-thioglucoside glucohydrolase; TGG) when tissue damage takes place. They form numerous breakdown products including isothiocyanates (ITCs; Wittstock and Burow, 2010), which are of foremost interest for their anticarcinogenic effects in humans (Satyan et al., 2006). The retention of GSLs in the postharvest storage period of rocket is therefore of critical importance for maximizing the potential health benefits for consumers (Martinez-Sanchez et al., 2006).

Salad rocket produces the ITC sulforaphane (SF; a breakdown product of 4-methylsulfinylbutyl GSL; glucoraphanin, GRA), which has been well documented for its potent anticarcinogenic properties (Herr et al., 2010). SF is abundant in broccoli, however its hydrolysis from GRA is often inhibited or prevented due to high cooking temperatures employed by consumers, which denatures myrosinase at temperatures >65°C (Rungapamestry et al., 2007).

A previous study by Bell et al. (2017b) observed that both GSL and ITC concentrations increased significantly in rocket salad post-processing, but that this varied according to cultivar. The study also highlighted that abundances at the point of harvest were not reflective of those found after 1 week of cold storage. The authors proposed that in response to the harvesting and washing process, stress responses within leaf tissues were initiated, leading to the increase in synthesis of GSLs and subsequent hydrolysis into ITCs. Sugar content, by comparison, showed little dynamic change and little reduction in the same samples, which could have implications for sensory perception and consumer acceptance (Bell et al., 2017a). For these reasons, GSLs and their breakdown products are of importance and interest to plant breeders and the scientific community.

Glucosinolates are synthesized as part of plant defense mechanisms against pests and diseases (Winde and Wittstock, 2011), and can also act as important S storage molecules (Kopriva et al., 2016). Compounds such as glucosativin (4-mercaptobutyl GSL; GSV) and glucorucolamine (4-cystein-S-yl-butyl GSL; GRL) are unique to the genera *Eruca* and *Diplotaxis* (“wild” rocket; Kim et al., 2007). GSV can exist in a dimer form (dimeric 4-mercaptobutyl GSL; DMB), and diglucothiobeinin [4-(β-D-glucopyranosyldisulfanyl)butyl GSL; DGTB] is a unique GSL dimer of these species (Bell et al., 2015). Despite the advances made in elucidating the *Arabidopsis thaliana* and *B. oleracea* GSL pathways, very little novel gene discovery has taken place outside of these species. The reason for this is the lack of genome sequence available for niche Brassicales species like *E. sativa*, and reliance upon knowledge about common compounds in related species, which is not able to account for the large differences observed in the GSL profile of rocket. Much is now known about the “core” GSL biosynthesis pathway in Arabidopsis and the regulatory mechanisms that respond to different biotic and abiotic stimuli (Francisco et al., 2016). Six main R2R3 MYB transcription factors (TFs) have been identified as regulators of GSL synthesis.

**FIGURE 1:**
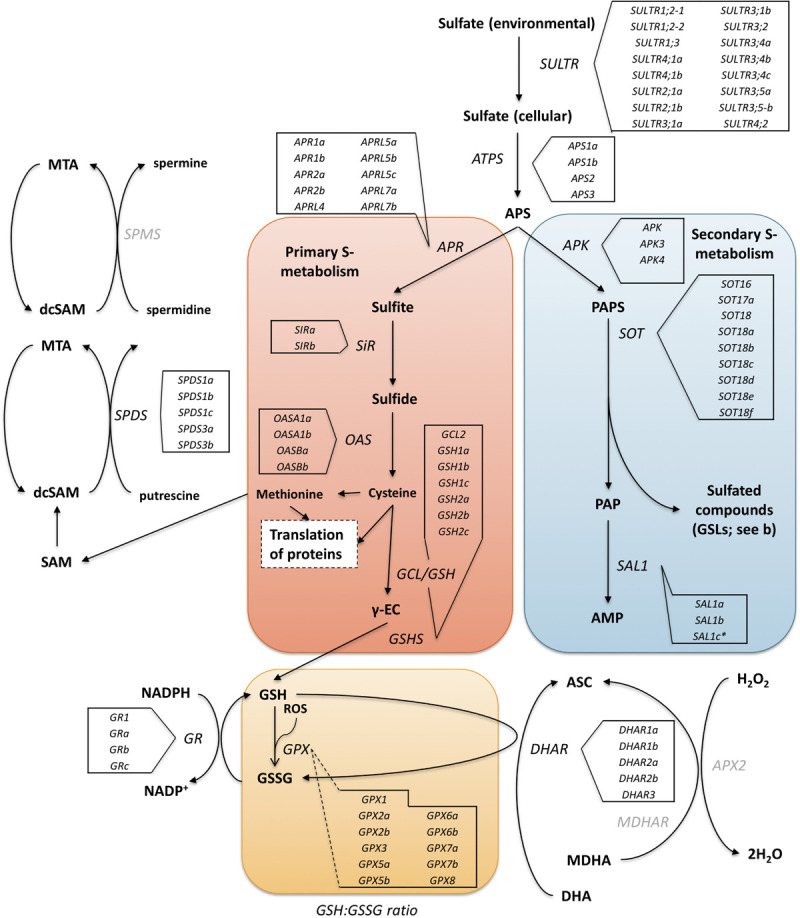
The primary (red box) and secondary (blue box) sulfur metabolism pathways of *A. thaliana* with identified homologous genes within the *Eruca sativa* genome annotation (see boxed insets) adapted from Chan et al. (2013). Environmental sulfur is assimilated and integrated into key amino acids (cysteine and methionine) and enzymes. Sulfur metabolism is also intrinsically linked with oxidative stress via glutathione synthesis. Under stress conditions 5′-phosphoadenosine-3′-phosphate (PAP), glutathione disulfide (GSSG), and reduced glutathione (GSH) direct sulfate toward GSH production. The GSH:GSSG redox state ratio is also known to influence sulfur assimilation rates (orange box). *SOT* (*sulfotransferase*) genes link secondary sulfur metabolism with the final sulfation step of GSL biosynthesis, and it is thought that *SAL1* plays an important role in regulating the activity of these genes through interaction with PAP. Genes with identified orthologs in the *E. sativa* genome annotation are written in black; those with no identified homologous sequence are written in gray. *SULTR*, *sulfate transporter*; ATP, adenosine triphosphate; *ATPS*, *ATP sulfurylase*; *APR*, *APS reductase*; *APK*, *APS kinase*; *SiR*, *sulfite reductase*; *OASTL*, *O-acetylserine lyase*; *GCL*, *glutamate cysteine ligase*; γ-EC, γ-glutamyl-cysteine; *GSHS*, *GSH synthetase*; *GR*, *glutathione reductase*; *GPX*, *glutathione peroxidase*; ASC, ascorbate; DHA, dehydroascorbate; *DHAR*, *DHA reductase*; MDHA, monodehydroascorbate; *MDHAR*, *MDHA reductase*; *APX2*, *ascorbate peroxidase 2*; H_2_O_2_, hydrogen peroxide; PAPS, 5′-phosphoadenosine-3′-phosphosulfate; SAM, S-adenosyl methionine; dcSAM, decarboxylated SAM; MTA, methylthioadenosine; *SPDS*, *spermidine synthase*; *SPMS*, *spermine synthase*. *Indicates a novel gene identification within the genome annotation.

Aliphatic GSLs are regulated by MYB28, MYB29, and MYB76 TFs, and indolic GSLs by MYB34, MYB51, and MYB122 (Frerigmann and Gigolashvili, 2014). These MYBs are in turn regulated by basic helix-loop-helix (bHLH) TFs such as MYC2, which are involved in plant defense response (Kazan and Manners, 2013). Other transcriptional regulators, such as *SLIM1* (*SULFUR LIMITATION 1*) and *SDI1* (*SULFUR DEFICIENCY INDUCED 1*) also interact with MYB TFs to regulate the use and efficiency of sulfur within the plant. As GSLs are a major sulfur sink (up to 30% of total plant S-content) the synthesis and catalysis of these compounds is crucial in times of stress (Figure 1; Chan et al., 2013; Aarabi et al., 2016).

Individual downstream GSL biosynthesis genes are regulated in response to a wide range of stress stimuli in response to changes in both MYB and MYC activity. Some of the most studied are genes encoding methylthioalkylmalate synthase (MAM) enzymes (regulators of GSL side chain lengths), genes encoding CYP79 enzymes (catalysts of the conversion of chain elongated amino acids into their respective aldoximes; Bell, 2019), and genes encoding CYP83 enzymes (that convert indolic aldoximes into corresponding thiohydroxymates; Bak and Feyereisen, 2001). Studies relating regulation to common horticultural practice, or transcriptomic regulation and response, are lacking. The effects of stresses imposed by harvesting, washing, processing, and storage differs between cultivars is not understood. *E. sativa* is a crop with great potential for enhancement of nutritional value, and it is therefore essential to understand how GSL biosynthesis and sulfur metabolism are regulated in order to direct breeding programs.

We present a *de novo E. sativa* reference genome sequence, and report on the specific effects harvest, wash treatment, and postharvest storage have on GSL biosynthesis and sulfur metabolism gene expression through RNA sequencing (RNAseq) in three elite inbred lines. We also present evidence of transcriptomic changes between first and second cuts of rocket plants, and how this in turn leads to elevated concentrations of both GSLs and ITCs. We hypothesized that each rocket line would vary in its ability to retain and synthesize GSLs post washing and during shelf life cold storage, as well as vary in their relative abundances between first and second cuts.

## Materials and Methods

### Plant Material for Genome Sequencing

Three elite inbred lines of salad rocket were produced through self-pollination for five generations at Elsoms Seeds Ltd. (Spalding, United Kingdom) from 2010 to 2016, giving an estimated inbreeding coefficient of 0.969 (Falconer and Mackay, 1996). Each line was derived from germplasm accessions obtained from the Leibniz-Institut für Pflanzengenetik und Kulturpflanzenforschung (IPK Gatersleben, Germany). For reasons of commercial sensitivity these lines (**A**, **B**, and **C**) and their lineage will not be identified.

For genome sequencing, plants of each line were grown under controlled growth room long-day cycle light conditions (200 μmol m^–2^ s^–^1; 22°C day, 15°C night) and watered as required. Leaf tissues were sampled and immediately frozen at −20°C. DNA was extracted using an E.Z.N.A. Plant DNA DS Mini Kit (Omega Bio-Tek, Norcross, GA, United States) in triplicate according to the manufacturer protocol, and sent to the Earlham Institute (Norwich, United Kingdom) for QC analysis. DNA samples for each line were pooled and quantified using a Qubit fluorometer and dsDNA assay kit (Thermo Fisher Scientific, Loughborough, United Kingdom) and assessed for quality using NanoDrop (Thermo Fisher Scientific). QC data for the sequenced DNA samples are provided in Supplementary Table 1.

### Genome Sequence Library Preparation and Assembly

*De novo* reference genome sequence was produced by interleaving Illumina MiSeq and HiSeq2500 sequence data (Illumina Inc., San Diego, CA, United States). DNA sequencing and assembly was performed as a service by the Earlham Institute. *De novo* genome sequencing and assembly was performed using PCR free paired-end (PE) and long mate pair (LMP) sequencing. After DNA sample QC, line **C** was selected for sequencing and reference genome assembly. One PCR free PE library was constructed from gDNA, and sequenced on one lane of an Illumina HiSeq2500 in rapid run-mode (v2) using 250 bp PE reads. LMP sequencing was also conducted using one set of Nextera libraries (Illumina) from gDNA, and sequenced on one lane of an Illumina MiSeq with 250 bp PE reads. After data QC and assembly of the high coverage PE library, LMP libraries were mapped to determine their suitability for assembly improvement. Three additional libraries were selected and re-sequenced to a higher depth of coverage on a single lane of an Illumina HiSeq2500 in rapid run-mode, to again yield 250 bp PE reads.

### Genome Sequencing Bioinformatics

FASTQ files were converted to BAM format using PicardTools (v1.84^1^; FastqToSam option) and then assembled using DISCOVAR *de novo* sequence assembler (build revision 52488; Weisenfeld et al., 2014). All LMP libraries were processed using NextClip (Leggett et al., 2014) to analyze and create a high quality read subset for scaffolding the DISCOVAR-assembled sequences. SOAP (Li et al., 2008) and SSPACE (Boetzer et al., 2011) were used to scaffold the DISCOVAR assembly using data from three of the NextClip-processed LMP read libraries.

### Genome Annotation

Annotation was performed by Novogene Co., Ltd. (Hong Kong). A homology and *de novo*-based approach was taken in order to identify TEs. The homology-based approach used known repetitive sequence databases: RepBase (Jurka et al., 2005), RepeatProteinMask, and RepeatMasker.^2^ *De novo* repeat libraries were created using LTR_FINDER (Xu and Wang, 2007), RepeatScout (see text footnote 2), and RepeatModeler.^3^

**FIGURE 2:**
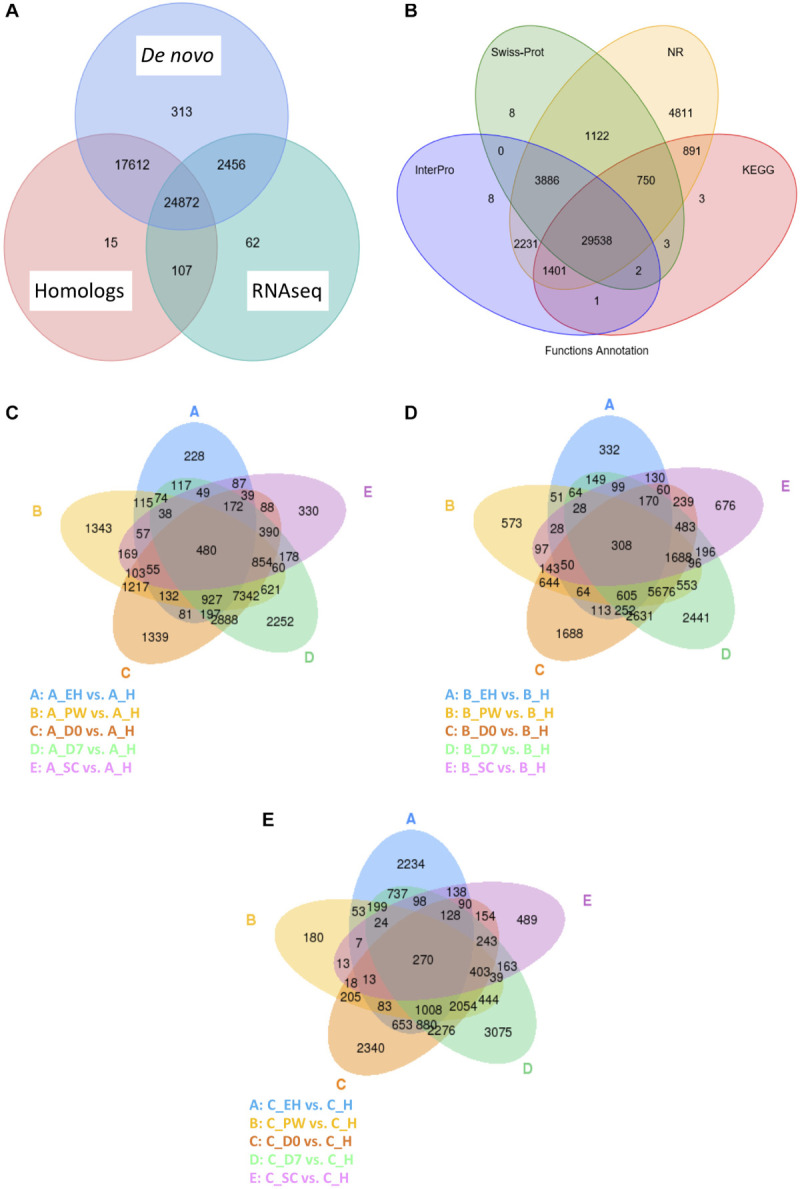
Venn diagrams of the *Eruca sativa* reference genome annotation gene identification sources **(A)** and functional annotation databases used to assign putative gene identities **(B)**. Also shown are Venn diagrams of global differentially expressed genes (DEGs) at an early harvest (**EH**), second harvest (**SC**), pre-wash (**PW**), post-wash (**D0**), and 7-day shelf life (**D7**) time points relative to a first harvest (**H**) time point of three elite breeding lines: **A (C)**, **B (D)**, and **C (E)**. The numbers of DEGs identified under each condition are contained within the ellipses and their overlaps.

An integrated approach was taken to compute consensus gene structures, such as cDNA, proteins in related species, and *de novo* predictions (Figure 2A). The homology-based approach used the related genomes of *Arabidopsis lyrata, A. thaliana, B. napus, Boechera stricta, Capsella rubella*, and *Raphanus sativus* to compare against *E. sativa* to find homologous sequences, and predict gene structures (using BLAST and genewise; Kent, 2002; Birney et al., 2004). *Ab initio* statistical models were also used to predict genes and their intron-exon structures; e.g., Augustus (Stanke et al., 2006), GlimmerHMM (Majoros et al., 2004), and SNAP.^4^ EVidenceModeler (EVM; Haas et al., 2008) software was then used to combine *ab initio* predictions, protein, and transcript alignments, and RNAseq data into weighted consensus gene structures. Lastly, PASA was used to update the consensus predictions by adding UTR annotations and models for alternative splicing isoforms. All predicted proteins were functionally annotated using alignments to SwissProt, TrEMBL (Bairoch and Apweiler, 2000), KEGG (Kanehisa and Goto, 2000), and InterPro (Zdobnov and Apweiler, 2001; Figure 2B).

The full reference genome sequence and annotation can be found in the European Nucleotide Archive (Assembly accession no: GCA_902460325; Study ID: PRJEB34051; Sample ID: ERS3673677; Annotation accession number ERZ1066251).

### Plant Material Growth and Collection for RNA, Elemental, and Phytochemical Analyses

Seeds were sown in a random order in seedling compost, and raised under controlled environment conditions in plastic trays inside a Weiss-Technik Fitotron cabinet (Weiss-Technik UK Ltd., Loughborough, United Kingdom). Daytime temperature was set to 20°C, and nighttime temperatures to 14°C (long day cycle; 16 h light, 8 h dark). Light intensity was set at 200 μmol m^–2^ s^–1^. During a 1-h period of “dawn” and “dusk,” light and temperature changes were ramped on a gradient. Humidity was ambient. After 10 days of growth, seedlings were transplanted to 1-L pots in standard peat-based compost.

Postharvest (post sample **H**), leaves were stored for 2 days in a cold store (4°C; Bell et al., 2017b). Samples for **D0** and **D7** were washed individually in mildly chlorinated water (sodium hypochlorite, 30 ppm; Suslow, 2000) for 2 min, then rinsed for 1 min with distilled water (all at >14°C to avoid cold-shock). Leaves were dried of excess moisture for 1 min using a kitchen salad spinner, then placed in fresh bags, sealed, and stored overnight at 4°C. Shelf life leaves were stored in the cold and dark (4°C) for 7 days (**D7**) – typical of the use-by date of commercially bagged leaves.

All samples were taken between the hours of 1–3 pm to mitigate diurnal fluctuations in phytochemical content and gene expression (Huseby et al., 2013). Immediately after each of the aforementioned samples was taken, leaves were frozen using liquid nitrogen and ground into a fine powder using a pestle and mortar. Samples were stored at −80°C in tubes and lyophilized prior to chemical analysis. A subset of non-lyophilized sample was kept aside for RNA extractions.

### RNA Extraction and Quality Control

RNA sequencing and bioinformatics was conducted on 18 plants from three elite inbred lines designated **A**, **B**, and **C**; giving a total of 54 plant samples. Time points corresponded to three harvest times (“early harvest” at 22 days after sowing, **EH**; “harvest” at 30 days after sowing, **H**; “second cut,” **SC**; leaves harvested from the same **H** plants 43 days after sowing), and three consecutive postharvest time points (harvested at 30 days after sowing and designated: “pre-wash,” **PW**; “day 0” of shelf life, 1 day post wash, **D0**; and “day 7” of shelf life, **D7**). See Supplementary Figure 1 for a schematic of the experimental design.

RNA for RNAseq and qRT-PCR analyses was extracted using RNeasy Plant Mini Kits (Qiagen, Manchester, United Kingdom) according to the manufacturer “Plants and Fungi” procedure. As part of the protocol, an on-column DNase digestion was incorporated according to the manufacturer RNase-Free DNase Set (Qiagen) protocol. Samples were checked for degradation and contamination prior to sequencing using agarose gel electrophoresis (1%, TAE buffer), Qubit, and NanoPhotometer (Implen, CA, United States) methods. Briefly, ≥2 μg of total RNA was obtained for each sample at a minimum concentration of ≥50 ng μL^–1^. RNA integrity was determined and evaluated using an Agilent 2100 Bioanalyzer (Fleige and Pfaffl, 2006). QC data for all RNA samples is provided in Supplementary Table 2.

### RNAseq Library Preparation and Sequencing

After QC procedures, sequencing libraries of three replicates were prepared using NEBNext Ultra RNA Library Prep Kit for Illumina (NEB, MA, United States) following the manufacturer’s instructions, and index codes were added to attribute sequences to each sample. mRNA was purified from total RNA by using poly-T oligo-attached magnetic beads. Fragmentation was carried out using divalent cations under elevated temperature in NEBNext First Strand Synthesis Reaction Buffer (5×). First strand cDNA was synthesized using random hexamer primer and M-MuLV Reverse Transcriptase (RNase H-). Second strand cDNA synthesis was subsequently performed using DNA Polymerase I and RNase H. Remaining overhangs were converted into blunt ends via exonuclease/polymerase activities. After adenylation of 3′ ends of DNA fragments, NEBNext Adaptor with hairpin loop structure were ligated to prepare for hybridization. In order to select cDNA fragments of 150–200 bp in length preferentially, the library fragments were purified with an AMPure XP system (Beckman Coulter, MA, United States). Three microliters USER Enzyme (NEB) was used with size-selected, adaptor-ligated cDNA at 37°C for 15 min, followed by 5 min at 95°C before PCR. PCR was performed with Phusion High-Fidelity DNA polymerase, Universal PCR primers and Index (X) Primer. Finally, PCR products were purified (AMPure XP system) and library quality was assessed using an Agilent 2100 Bioanalyzer system.

The clustering of the index-coded samples was performed on a cBot Cluster Generation System using HiSeq PE Cluster Kit cBot-HS (Illumina) according to the manufacturer’s instructions. After cluster generation, the library preparations were sequenced on an Illumina Hiseq platform and 125/150 bp PE reads were generated.

### RNAseq Bioinformatics

Raw data (raw reads) of FASTQ format were firstly processed through Novogene Co., Ltd., in-house perl scripts. Clean reads were obtained by removing reads containing adapter, reads containing ploy-N, and low quality reads from the raw data. Q20, Q30, and GC content of the clean data were calculated.

An index of the reference genome was built using Bowtie (v2.2.3), and PE clean reads were aligned to the reference genome using TopHat (v2.0.12; Langmead et al., 2009; Anders et al., 2010; Langmead and Salzberg, 2012). TopHat was selected as the mapping tool as it can generate a database of splice junctions based on the gene model annotation file, and thus a better mapping result is achieved than other non-splice mapping tools.

HTSeq (v0.6.1) was used to count the read numbers mapped to each gene (Trapnell et al., 2010). FPKM (Fragments Per Kilobase of transcript sequence per Millions base pairs sequenced) of each gene was calculated based on the length of the gene and reads count mapped to each gene. Differential expression analysis of each sample point/inbred line (three biological replicates) was performed using the DESeq R package as described by Anders et al. (2010; 1.18.0). After normalization, the resulting *p*-values were adjusted using Benjamini and Hochberg’s approach for controlling the false discovery rate (*q*-value). Genes with a *q*-value < 0.05 were assigned as being significantly differentially expressed.

### RNAseq Validation by qRT-PCR

Independent RNA extractions were conducted for qRT-PCR validation, and quality checked according to the same protocols and instrumentation as for RNAseq. cDNA synthesis was conducted using qPCRBIO cDNA Synthesis Kit (PCR Biosystems Ltd., London, United Kingdom) according to the manufacturer instructions. cDNA was then diluted 10× prior to analysis. All 54 biological samples were tested in triplicate.

PCR primers were designed using PRIMER3^5^ using default settings. Ten genes related to GSL biosynthesis and transcription were selected at random for the validation analysis (*BCAT4, CYP83B1, MYB122-1a, MYB51a, SOT16, SUR1, TGG1b, TGG1d, TGG1j*, and *UGT74B1*), with *ACT11* used as a reference gene (Hu et al., 2009). Gene sequences of *E. sativa* were obtained using NovoFinder (Novogene Co., Ltd.), and primer annealing sites were designed to span intron–intron boundaries where possible (see Supplementary Table 3).

Analysis was performed using the 2^–ΔΔ^ method (Livak and Schmittgen, 2001) on a Roche LightCycler 480 Instrument and the manufacturer Advanced Relative Quantification protocol (v1.5.1). Primer efficiencies were determined by analyzing each primer set with log-fold dilutions of cDNA (Supplementary Table 3). 2× qPCRBIO SyGreen Blue Mix Lo-ROX (PCR Biosystems Ltd.) was used to prepare a master mix for all reactions. Reaction volumes totaled 10 μL, and the PCR method used was as per the manufacturer recommendations.

Data were normalized and expressed as the log2-fold change relative to *ACT11*. RNAseq data for each of the tested genes were similarly converted for direct comparison of the two methodologies (Supplementary Figure 3). An ANOVA test found no significant difference between the two data sets.

### Co-expression Module Identification and Gene Set Enrichment Analysis

Full gene expression data of lines **A, B**, and **C** were analyzed using the webCEMiTool (Co-Expression Module Identification Tool) pipeline (Russo et al., 2018; Cardozo et al., 2019). A variance filter value of 0.01 was used to ensure the highest level of statistical stringency. RNAseq normalization mean variance dependencies were corrected using the Variance Stabilizing Transformation (VST) option. Pearson’s correlation method was selected for identification of the gene modules. As part of the pipeline, a Gene Set Enrichment Analysis (GSEA) was performed using each module as a gene set. A Normalized Enrichment Score (NES) was generated for each phenotype, as well as a Benjamini-Hochberg *q*-value.

### Intact Glucosinolate Extraction and Analysis by LC-MS

Intact GSLs were extracted according to the protocol used by Bell et al. (2015). Immediately before LC-MS analysis, samples were diluted with 4 mL of HPLC-grade water. Samples were analyzed in a random sequence with standards and QC samples. External standards of sinigrin (SIN; >99%, TLC), GRA (99.86%, HPLC), glucoalyssin (GAL; 98.8%, HPLC), 4OHB (96.19%, HPLC), and GER (99.68%, HPLC) were prepared for quantification of GSL compounds. SIN was used to quantify DGTB, GSV, and DMB, as no standards are available for these compounds. 4OHB was used to quantify the indole GSLs 4MOB and neoglucobrassicin (NGB). All standards with the exception of SIN (Sigma Merck, Gillingham, United Kingdom) were purchased from PhytoPlan (Heidelberg, Germany). Limits of detection (LOD) and quantification (LOQ) were established for the method by running serial dilutions of SIN (LOD = 2.14 mg.kg^–1^; LOQ = 6.48 mg.kg^–1^).

LC-MS analysis was performed in the negative ion mode on an Agilent 1260 Infinity Series LC system (Agilent, Stockport, United Kingdom) equipped with a binary pump, degasser, auto-sampler, column heater, and diode array detector, coupled to an Agilent 6120 Series single quadrupole mass spectrometer. Separation of samples was achieved on a Gemini 3 μm C_18_ 110Å (150 × 4.6 mm) column (with Security Guard column, C_18_; 4 mm × 3 mm; Phenomenex, Macclesfield, United Kingdom). GSLs were separated during a 40 min chromatographic run, with a 5 min post-run sequence. Mobile phases consisted of ammonium formate (0.1%; A) and acetonitrile (B) with the following gradient timetable: (i) 0 min (A–B, 95:5, v/v); (ii) 0–13 min (A–B, 95:5, v/v); (iii) 13–22 min (A–B, 40:60, v/v); (iv) 22–30 min (A–B, 40:60, v/v); 30–35 min (A–B, 95:5, v/v); (v) 35–40 min (A–B, 95:5, v/v). The flow rate was optimized for the system at 0.4 mL min^–1^, with a column temperature of 30°C, and 20 μl of sample injected. Quantification was conducted using a diode array detector at a wavelength of 229 nm.

MS settings were as follows: Atmospheric pressure electrospray ionization was carried out in negative ion mode (scan range m/z 100–1500 Da). Nebulizer pressure was set at 50 psi, gas-drying temperature at 350°C, and capillary voltage at 2,000 V. Compounds were identified using their primary ion mass (M-H)^–^, and comparison to authentic standards (Cataldi et al., 2007; Lelario et al., 2012). Data were analyzed using Agilent OpenLAB CDS ChemStation Edition for LC-MS (vA.02.10). GSL concentrations from each time point were averaged over three biological replicates with two technical replicates of each (*n* = 6). This approach was also conducted for glucosinolate hydrolysis product (GHP) and monosaccharide content.

### Glucosinolate Hydrolysis Product Extraction and Analysis by GC-MS

GHPs were extracted according to the protocol presented by Ku et al. (2016) with the following modification: samples were hydrolyzed in d.H_2_O for 3 hours at 30°C before extraction with dichloromethane (DCM) for 21 h. This duration was optimized for maximum yields of GHPs by comparison of extraction times: 3 h incubation in d.H_2_O at 30°C with immediate DCM extraction; 3 h incubation in d.H_2_O at 30°C, with three, nine, and 21 h post incubation with DCM.

GC-MS analysis and GHP identification was conducted according to the method presented by Bell et al. (2017b). Concentrations of all GHPs were calculated as equivalents of SF standard (Sigma).

### Monosaccharide Extraction and Analysis by HPLC

Free monosaccharides were extracted according to the method presented by Bell et al. (2017a), with the exception that 0.2 g of lyophilized leaf powder was extracted. Extracts were analyzed on an Agilent 1100 series HPLC system equipped with a binary pump, degasser, and auto-sampler, with an external column heater (50°C). A Bio-Rad Aminex HPX-87H (300 × 7.8 mm, 9 μm particle size) column with a Micro-Guard Cation H guard column (Bio-Rad, Watford, United Kingdom) was used to achieve separation with an isocratic gradient of 5 mM sulfuric acid, and a flow rate of 0.6 mL per min. A Polymer Laboratories ERC-7515 refractive index detector (Church Stretton, United Kingdom) was used to detect monosaccharides. Compounds were quantified using authentic standards and analyzed with Agilent ChemStation software (Santa Clara, CA, United States).

### Sulfur Content Analysis by ICP-OES

Lyophilized samples were weighed into acid washed glass boiling tubes, and pre-digested in 70% nitric acid for 24 h, before being heated to 90°C for 2 h using a heat block. Once cooled, these were filtered through a 0.45 μM syringe filter, and diluted to give an acid concentration of 3%. These samples were analyzed using Inductively Coupled Plasma-Optical Emission Spectroscopy (ICP-OES; Perkin Elmer Optima 7300 DV). Sulfur content was determined using the radial signal at 181.975 nm. Due to the small plant size and limited amounts of dried leaf powder, **EH** samples were not included in sulfur content analysis.

### Statistical Analyses

All statistical analyses (not included in bioinformatics sections) were performed using XL Stat (Addinsoft, Paris, France). Shapiro–Wilk normality tests were conducted for all variables, all of which were concluded to fit a normal distribution. ANOVA with *post hoc* Tukey’s Honest Significant Difference (HSD) tests were performed to generate multiple pairwise comparisons between sampling points for each cultivar (i.e., **H** vs. **D7** for cultivar **B**) and between cultivars at each respective time point (i.e., **A** vs. **B** for time point **H**) for phytochemical and elemental data (Supplementary Data File 1). Principal Component Analysis (PCA) was performed using Pearson correlation coefficient analysis, *n*−1 standardization, Varimax rotation, and Kaiser Normalization. Phytochemical data were regressed onto the gene expression data as supplementary variables for the targeted analysis.

## Results

### *Eruca sativa* Genome Assembly and Annotation

Elite breeding line **C** was assembled into 49,933 contigs (≥500 bp). This line was chosen as the reference sequence because of its higher DNA concentration and optimal 260/280 ratio (Supplementary Table 1). The resulting assembly was ∼851 Mb in size (Table 1). Transposable elements (TEs) within the *E. sativa* genome comprise 66.3% of its content. The majority of TEs are long terminal repeat (LTR) retrotransposons (37.3%), with long interspersed nuclear elements (LINEs; 3.3%) and short interspersed nuclear elements (SINEs; 0.3%) having lower relative abundance. A total of 18.2% of all TEs identified were of unknown classification (Table 2).

**TABLE 1:** Summary of genome assembly and annotation of *Eruca sativa*.

**Genome assembly**	**0 bp**		**1,000 bp**		**Largest contig**		**Total (500 bp)**	
Contig number	1,041,818		12,352		1,477,633		49,933	
Total length	850,956,505		562,271,846				586,731,295	
**Assembly related statistics**								
**GC%**	**N50**	**NG50**	**N75**	**NG75**	**L50**	**LG50**	**L75**	**LG75**

36.25	196,831	136,378	87,576	2,634	789	1,256	1,889	7,243

**TABLE 2:** Transposable elements content in the reference genome.

**Type**	*De novo* + Repbase*		**TE proteins^$^**		**Combined TEs^ε^**	
	**Length (bp)**	**% in genome**	**Length (bp)**	**% in genome**	**Length (bp)**	**% in genome**
DNA	69,251,054	8.14	21,607,510	2.54	76,517,426	8.99
LINE	20,290,781	2.38	17,153,783	2.02	28,200,567	3.31
SINE	2,134,305	0.25	0	0	2,134,305	0.25
LTR	311,377,915	36.59	91,001,347	10.69	317,124,290	37.27
Other∧	106,176	0.01	0	0	106,176	0.01
Unknown∧∧	155,033,031	18.22	0	0	155,033,031	18.22
Total	547,675,259	64.36	129,571,414	15.23	563,873,839	66.26

*RepeatMasker based on the uclust algorithm combined with the known Repbase and *de novo* repeat library created by RepeatModeler/Repeat Scout/LTR_finder. ^$^RepeatProteinMask based on Repbase. ^ε^The non-redundant set of results combining *De novo* + Repbase TEs and TE proteins. ∧Repeats that can be classified by RepeatMasker, but not included by classes above. ∧∧Repeats that could not be classified by RepeatMasker.

A combined method of *de novo* prediction, RNAseq (of leaf, stem, and root tissue), and homology with related species’ genomes was used to predict gene numbers in *E. sativa*. A total of 45,438 protein-coding genes were identified within the assembly, with an average length of 1,889.6 bp, and an average of 4.8 exons per gene. This genome size is smaller than that predicted for radish (*R. sativus*), and larger than *A. lyrata* (Table 3), and is consistent with what is known of Brassicales phylogeny (Arias and Pires, 2012). A total of 98.3% of predicted genes were found to have homology with other plant species (Figure 2B and Supplementary Table 4). The average coding sequence (CDS) length of genes identified in rocket was 1,069.4 bp, which is most similar to that found in *A. lyrata*. Average intron and exon lengths were 224.8 and 218.3 bp, respectively; which is most similar to the *A. lyrata* genome.

**TABLE 3:** Predicted protein-coding genes within the *E. sativa* reference genome.

**Gene set**		**Number**	**Average gene length (bp)**	**Average CDS length (bp)**	**Average exons per gene**	**Average exon length (bp)**	**Average intron length (bp)**
De novo*	Augustus	50,179	1,701.56	1,024.89	4.48	228.88	194.57
	Glimmer HMM	73,989	1,335.36	725.53	3.03	239.16	299.86
	SNAP	80,264	1,231.13	728.3	4.02	180.98	166.27
	Geneid	100,165	2,127.25	585.82	3.07	191.03	745.87
	Genscan	71,813	3,942.04	785.32	3.92	200.11	1,079.4
Homolog∧	*Arabidopsis lyrata*	32,667	1,867.18	1,084.09	4.86	223.12	202.93
	*Arabidopsis thaliana*	27,416	1,870.34	1,218.4	5.13	237.58	157.91
	*Brassica napus*	101,040	1,764.75	1,001.16	4.91	204.06	195.48
	*Boechera stricta*	27,416	2,006.68	1,181.2	5.09	231.86	201.61
	*Capsella rubella*	26,521	1,958.82	1,248.6	5.19	240.53	169.46
	*Raphanus sativus*	49,733	2,064.75	1,194.41	4.94	241.57	220.66
RNAseq∧∧	Cufflinks	43,200	2,848.16	1,723.58	6.03	285.65	223.41
	PASA	37,870	1,744.08	1,034.72	4.77	216.9	188.13
EVM		59,643	1,665.12	926.2	4.17	222.2	233.23
PASA-update		59,491	1,656.25	929.33	4.17	222.9	229.36
Final set		45,438	1,889.6	1,069.44	4.76	224.81	218.3

*The combined results by EVM of *5-ab initio* gene predictions. ∧The combined results by EVM of homology-based gene prediction. ∧∧The combined results by EVM of transcriptome data sets.

### RNAseq Analysis of *E. sativa* Plants

#### Global Differential Gene Expression

After sample QC and clean-up over 2.6 billion clean PE reads were produced, averaging ∼49 million reads per sample. Q20 (<1% error rate) averaged 96.3%, Q30 (<0.1% error rate) averaged 90.8%, and GC content ranged from 44.5 to 47.4%.

The total numbers of differentially expressed genes (DEGs) for line **A**, **B**, and **C** are presented in Figures 2C–E, respectively. Few significant DEGs were observed between **EH** and **H** samples for lines **A** and **B** (<333; Figures 2C,D), whereas they were observed at a higher rate in **C** (2,234; Figure 2E). This indicates a high degree of plasticity of **C** across growth stages.

This trend was reversed at **PW**, where 180 DEGs were observed compared to **H** in **C**, and 1,343 were observed in **A**. During shelf life (**D0** and **D7**) **C** expressed a greater number of DEGs compared to **H**, than **A** or **B** (2,340 at **D0** and 3,075 at **D7**, respectively). By contrast, DEGs at **SC** were much less variable between the three lines (330–676) indicating a greater degree of uniformity of expression in the second cut.

**FIGURE 3:**
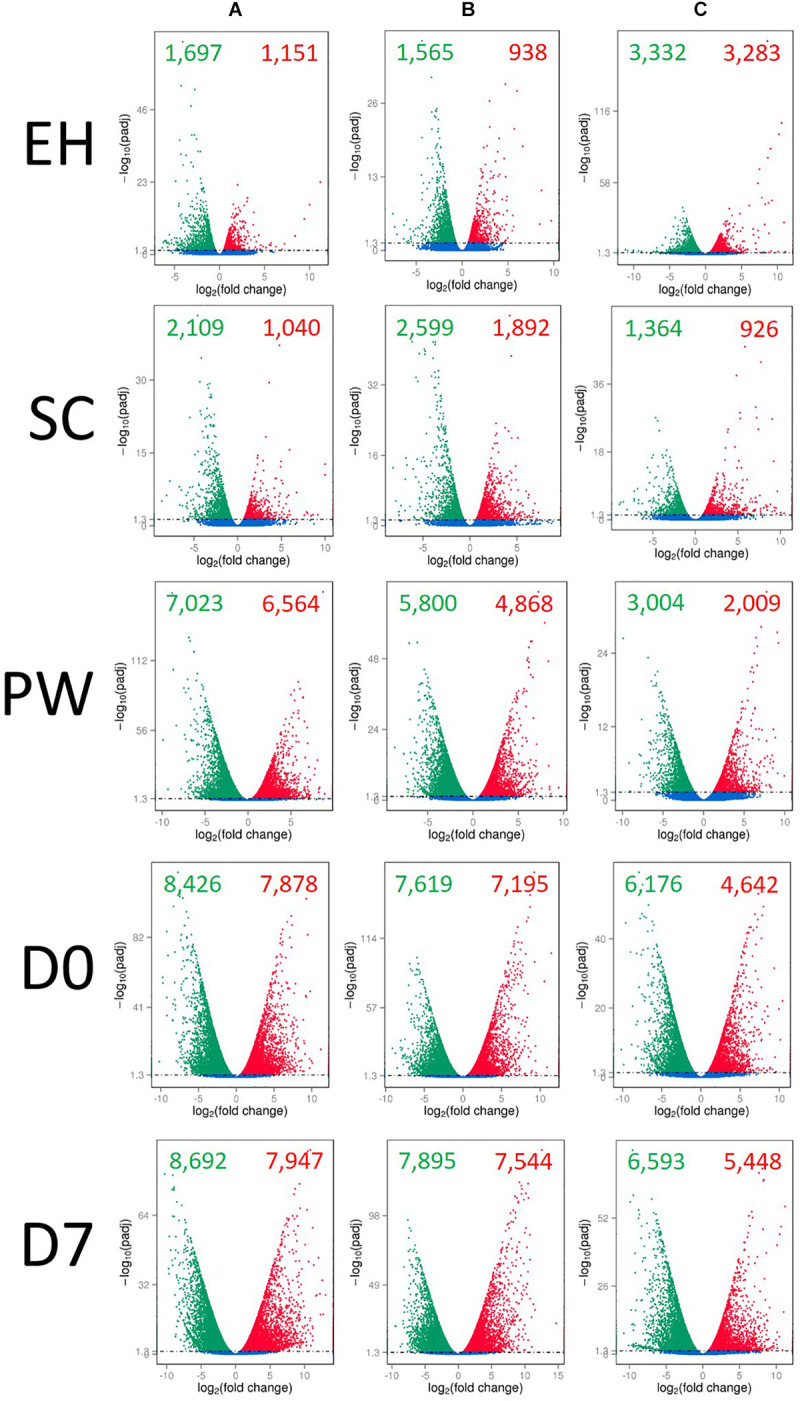
Global differential gene expression and distribution in three elite inbred lines of *Eruca sativa* (**A–C**) across five time points, relative to harvest (**H**) samples for each line. Individual volcano plots display the log2-fold change of gene expression on the *x*-axis, and the degree of statistical significance on the *y*-axis (*q*-value < 0.05, with removal of variations between biological replicates). Red dots, significantly up-regulated genes (relative to **H**); green dots, significantly down-regulated genes (relative to **H**); blue dots, no significant change in expression. **EH**, early harvest; **SC**, second harvest; **PW**, pre-wash; **D0**, post-wash; and **D7**, 7-day shelf life.

Up and down regulation of global DEGs relative to **H** are presented in Figure 3. Several differences between the lines are illustrative of the complex and varied responses genotypes have when grown under the same environmental conditions. Lines **A** and **B** have very similar up/down expression patterns of genes at ontogenic (**EH** and **SC**) and postharvest (**D0**, and **D7**) time points. The exception to this was at **PW**, where line **A** showed higher numbers of down (7,023) and up (6,564) regulated genes than **B** (5,800 down; 4,868 up) relative to their respective numbers at **H**. The largest differences in up/down expression patterns can be seen for line **C**. At **EH**, more than double the numbers of genes were up/down regulated relative to **H**, compared to lines **A** and **B**. At **SC**, the opposite was observed, suggesting that second cuts of line **C** are more similar to first cuts (**H**) than **A** and **B**, in terms of their gene expression. This is of relevance to growers and breeders, as it indicates that greater transcriptional and metabolic consistency may be achievable between successive cuts of rocket if an appropriate cultivar is selected. This trend was also observed postharvest, where line **C** had several thousand fewer genes up/down regulated than **A** and **B**. As will be discussed in following sections, the apparently reduced transcriptional response to stimuli, such as harvesting, processing, and cold storage, may be indicative of greater resilience to stress in line **C**.

#### Co-expression and Gene Set Enrichment Analysis

**FIGURE 4:**
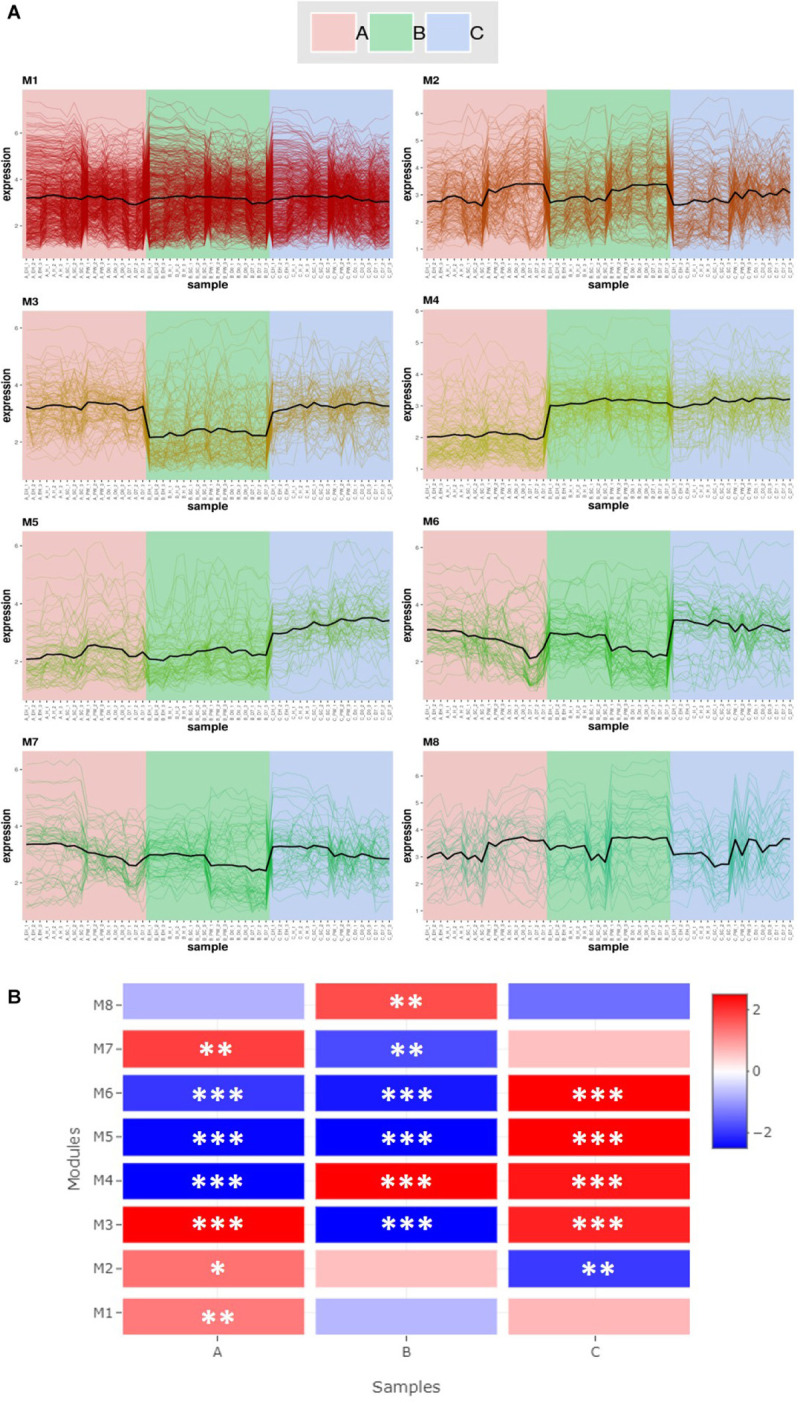
Co-expression module analysis **(A)** and Gene Set Enrichment Analysis (GSEA; **(B)** of three elite inbred lines of *Eruca sativa*. Eight gene modules (M1–M8) were identified within the transcriptomes of lines **A**, **B**, and **C** (see inset for color coding). Modules contain 583 (**M1**), 184 (**M2**), 112 (**M3**), 107 (**M4**), 72 (**M5**), 65 (**M6**), 58 (**M7**), and 38 (**M8**) genes, respectively **(A)**. The heat-map **(B)** illustrates differences in module activity (red, high; blue, low) between each genotype, and asterisks indicate the level of Normalized Enrichment Score (NES) significance: *p ≤ 0.05; **p ≤ 0.01; ***p ≤ 0.001. Complete gene lists for each module and the corresponding enrichment analysis statistics are presented in Supplementary Data File 2.

Co-expression analysis of RNAseq data produced eight gene modules (Figure 4). These contained 583 (**M1**), 184 (**M2**), 112 (**M3**), 107 (**M4**), 72 (**M5**), 65 (**M6**), 58 (**M7**), and 38 (**M8**) genes, respectively (Figure 4A). Figure 4B shows that there are distinct and significant module expression patterns for each of the three genotypes (**A**, **B**, and **C**). Line **A** had significant positive expression of modules **M1**, **M2**, and **M7** in comparison to the other two lines. Line **C** had significantly greater expression in modules **M5** and **M6**, and line **B** in module **M8**. These patterns are most distinct in the Figure 4A plots for **M3**, **M4**, and **M5**; but there are also subtle differences within genotypes where there are distinct increases/decreases for the postharvest time points (**PW**, **D0**, and **D7**). This can be most clearly seen in **M2**, **M6**, **M7**, and **M8**.

The genes contained within each respective module are listed in Supplementary Data File 2. Several of these modules contain genes related to the GSL biosynthesis pathway, as well as myrosinases. Module **M1** is the by far the largest and contains “hub” genes related to photosynthesis (*LHCA4* and *PSAH2*). It contains a number of indole GSL biosynthesis genes, such as *CYP79B2a, CYP83B1, SOT16, CYP81F2b, IGMT1a*, and *IGMT4a*. Two TGG1 myrosinase genes are also present (*TGG1b* and *TGG1e*) as well as two putative *TGG6* genes. This module had significantly higher expression in line **A** than the two others (Figure 4B). Of note in **M2** is the presence of the TF *HY5*, which as previously been associated with the diurnal regulation of GSL biosynthesis in *A. thaliana* (Huseby et al., 2013).

Contained within **M6** are two genes pertinent to sulfur assimilation and aliphatic GSL biosynthesis; *SiRa* (sulfite reductase) and *MYB28b*. MYB28 has been shown to upregulate expression of SiR in Arabidopsis (Sønderby et al., 2007) as well as other genes in the sulfur metabolism pathway.

**M8** is the smallest of the gene modules identified, but has a notable hub gene related to senescence: *STR15*, also known as *SENESCENCE1* (*SEN1*). This gene has been previously linked with expression of defense-related signaling pathways (Schenk et al., 2005) and increases in senescence-induced oxidative stress (Hye et al., 2004). Also of note within this module is *OXS3* (*OXIDATIVE STRESS 3*), which is part of cellular oxidative stress response (Blanvillain et al., 2009). This module had significantly higher expression in line **B** relative to **A** and **C** (Figure 4), and as will be discussed, may contribute to differences in observed shelf life GSL phenotypes.

### Sulfur Assimilation and Glucosinolate Biosynthesis Pathway Gene Expression

#### Sulfate Assimilation Gene Expression

**FIGURE 5:**
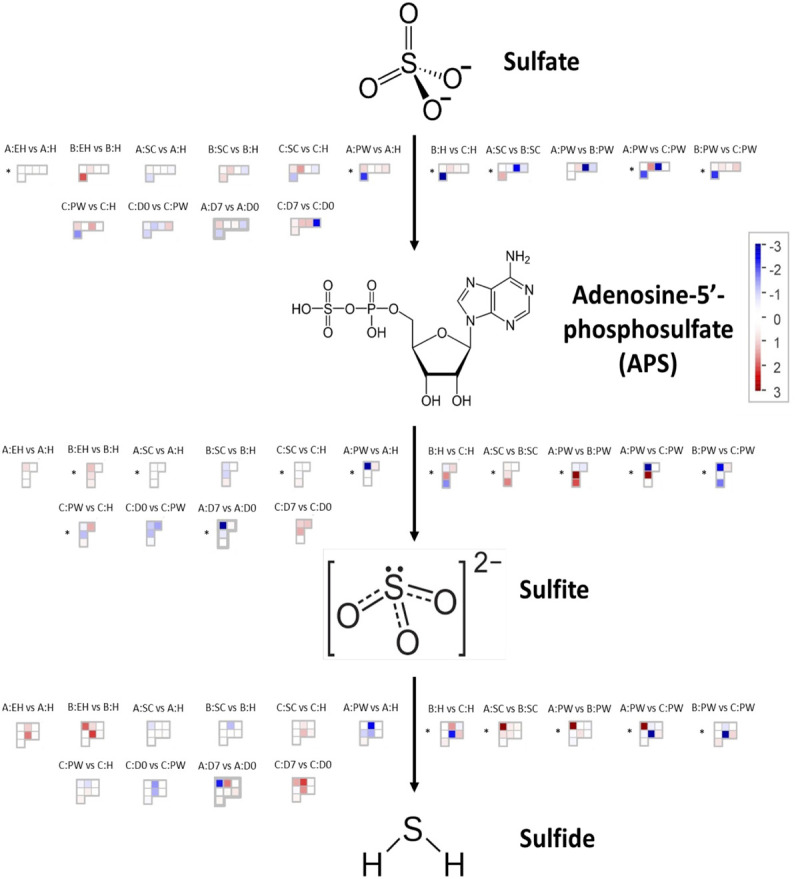
RNAseq expression data (FPKM) for genes involved with sulfate assimilation in three elite inbred lines of *Eruca sativa*. A custom MapMan (version 3.6.0RC1) annotation file of the *E. sativa* reference genome was created using Mercartor4 (version 1.0, plaBi dataBase, Institute of Biology, Aachen, Germany), and used to visualize differential expression of genes within the sulfate assimilation pathway. Asterisks denote significance of up/down regulation. **EH**, early harvest; **H**, harvest; **SC**, second harvest; **PW**, pre-wash; **D0**, post-wash; and **D7**, 7-day shelf life.

Figure 5 presents differential gene expression within the sulfate assimilation pathway of *E. sativa*. All significances quoted hereafter were at the *q* < 0.05 significance level. In the primary stages of sulfur metabolism, sulfate is activated via adenylation to adenosine-5′-phosphosulfate (APS), catalyzed by ATP sulfurylase (ATPS; Anjum et al., 2015). In *E. sativa* four ATPS-encoding genes were identified: *APS1a, APS1b, APS2*, and *APS3* (Figure 1). Very few significant DEGs were observed between sample points for each respective rocket line (see Supplementary Data File 3 for full values and statistics of each sample comparison). However, between **H**, **SC**, and **PW**, each respective line did show significant differential expression of ATPS genes.

**FIGURE 6:**
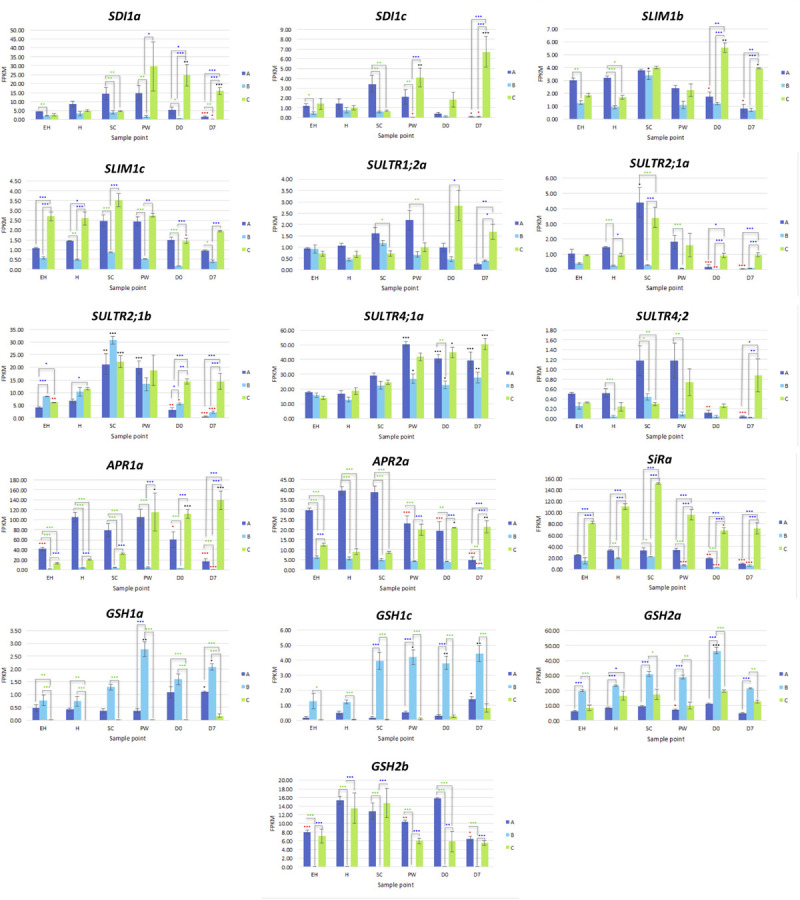
RNAseq expression data (FPKM) for genes involved with sulfate transport and redox response in three elite inbred lines of *Eruca sativa* (**A**, dark blue; **B**, light blue; **C**, green). Standard errors of the mean expression values are represented by error bars. Asterisks denote levels of significance of up and down regulation within sample points (between each inbred line) and relative to the point of harvest for each respective sample point: *p ≤ 0.05; **p ≤ 0.01; ***p ≤ 0.001; green, significant up regulation between lines **A**, **B**, and **C**; blue, significant down regulation between lines **A**, **B**, and **C**; black, significant up regulation relative to **H**; red, significant down regulation relative to **H**. **EH**, early harvest; **H**, harvest; **SC**, second harvest; **PW**, pre-wash; **D0**, post-wash; and **D7**, 7-day shelf life.

In the second stage of the pathway, APS is reduced to sulfite by adenosine-5′-phosphosulfate reductase (APR; Capaldi et al., 2015). Four APRs were identified (*APR1a, APR1b, APR2a*, and *APR2b*) as well as six APR-like genes (*APRL4, APRL5a, APRL5b, APRL5c, APRL7a*, and *APRL7b*). *APR1a* and *APR2a* showed significant differential expression across multiple samples and time points (Figure 6). Line **B** displayed low relative expression of these genes compared to **A** and **C**. Line **C** exhibits significantly higher expression postharvest compared to **H**; 2.2 log2-fold (**D0**) and 2.7 log2-fold (**D7**) increases of *APR1a*, and 0.9 log2-fold (**D0**) and 1.1 log2-fold (**D7**) increases of *APR2a* were observed. We hypothesize that this may be indicative of a greater ability to assimilate sulfate via APR enzymes to facilitate and maintain secondary metabolite biosynthesis for longer into shelf life.

Two copies of genes encoding sulfite reductase (SiR; *SiRa* and *SiRb*) were identified. *SiRa* showed significantly higher levels of expression in line **C** (Figure 6). Line **C** had no significant change in activity of this gene relative to time point **H**, however both lines **A** and **B** had significantly lower expression postharvest (Figure 6).

#### Sulfur Metabolism Transcription, Regulation, and Transport Gene Expression

Three copies of SDI1 (*SDI1a, SDI1b*, and *SDI1c*) and three copies of SLIM1 (SULFUR LIMITATION 1, aka ETHYLENE INSENSITIVE-like 3; *SLIM1a, SLIM1b*, and *SLIM1c*) were identified within the genome annotation. These genes are thought to play critical roles in the management and use-efficiency of sulfur in plants, and have been linked with optimization of GSL biosynthesis under S-limited conditions in *A. thaliana* (Aarabi et al., 2016).

*SDI1a* and *SDI1c* were differentially expressed between each line (Figure 6 and Supplementary Data File 3), with **C** having the highest levels of expression postharvest. It might be expected that each line would see a similar trend of expression over the course of shelf life, as additional sulfur is not obtainable; however only line **C** displayed this (Figure 6).

*SLIM1b* was significantly higher at time points **D0** and **D7** relative to **H** (a 1.4 and 1.1 log2-fold significant increase, respectively) in line **C**. Expression of *SLIM1c* by comparison was not significantly different for each respective plant line between time points, but there were clear and significant differences in expression between lines (Supplementary Data File 3). Line **C** had highest expression of this gene, followed by **A**; with **B** having significantly lower expression overall (Figure 6). Previous studies have shown that SLIM1 down regulates APK gene expression and GSL biosynthesis as a way of conserving sulfur for primary metabolism (Chan et al., 2019). Our data suggest that this is only the case between *SLIM1a* and *APK3* (*r* = −0.597, *q* < 0.001; Supplementary Data File 1). *SLIM1a* expression was positively (and significantly) correlated with *APK* expression (*r* = 0.521), and *SLIM1b* and *SLIM1c* with *APK4* (*r* = 0.575 and 0.698, respectively; Supplementary Data File 1). This suggests *E. sativa* has a complex and interacting network of sulfur metabolism genes, where functions may not necessarily be analogous to those found in *A. thaliana*.

Sixteen sulfur transport (*SULTR*) genes were identified within the annotation; of note were *SULTR1;2a, SULTR2;1a, SULTR2;1b, SULTR4;1a*, and *SULTR4;2*. *SULTR1;2a* has been associated with the uptake of environmental sulfate in root tissues (Supplementary Data File 3), but we detected low levels of expression in leaf tissues. Postharvest, line **C** had differential expression of this gene compared to **A** and **B** in **D7** samples (Figure 6). This was more pronounced for *SULTR2;1a* and *SULTR2;1b*, and both **A** and **B** had significant reductions in expression at **D0** and **D7** relative to **H**. **SC** samples showed significant increases relative to **H**, with the exception of *SULTR2;1a* in **B**.

*SULTR4;1* and *SULTR4;2* genes also had distinct patterns of expression between lines. *SULTR4;1a* saw significant increases in expression in postharvest samples relative to **H** (Figure 6). Line **A** had higher expression of *SULTR4;2* during growth before declining significantly post-wash (**D0**). The opposite trend was seen in **C**, where gene expression peaked at **D7**. These data are suggestive of more active intra-leaf sulfur transport in line **C** postharvest, and may be associated with the higher expression of APR, SiR, SDI1, and SLIM1 genes to facilitate more efficient S utilization during this period.

#### Glutathione Synthesis

With the exception of *GSH2b*, glutathione synthetase genes were most highly expressed in rocket line **B**, with significant increases observed postharvest (Figure 6). Lines **A** and **C** were unchanged between sample points for these genes, but had a marked difference in expression for *GSH2b* relative to each other. **B** had negligible levels of *GSH2b* expression.

As both glutathione and secondary S-containing metabolites, such as GSLs, have been associated with antioxidant responses (Chan et al., 2019) the differences observed between each of the lines in terms of both GSL concentrations and glutathione-related gene expression, may be indicative of different adaptive metabolic strategies for dealing with oxidative stress postharvest. Lines **A** and **C** favor secondary sulfur metabolism and the synthesis of GSLs, and **B** favors primary sulfur metabolism and glutathione synthesis.

#### Glucosinolate-Related Transcription Factors

**FIGURE 7:**
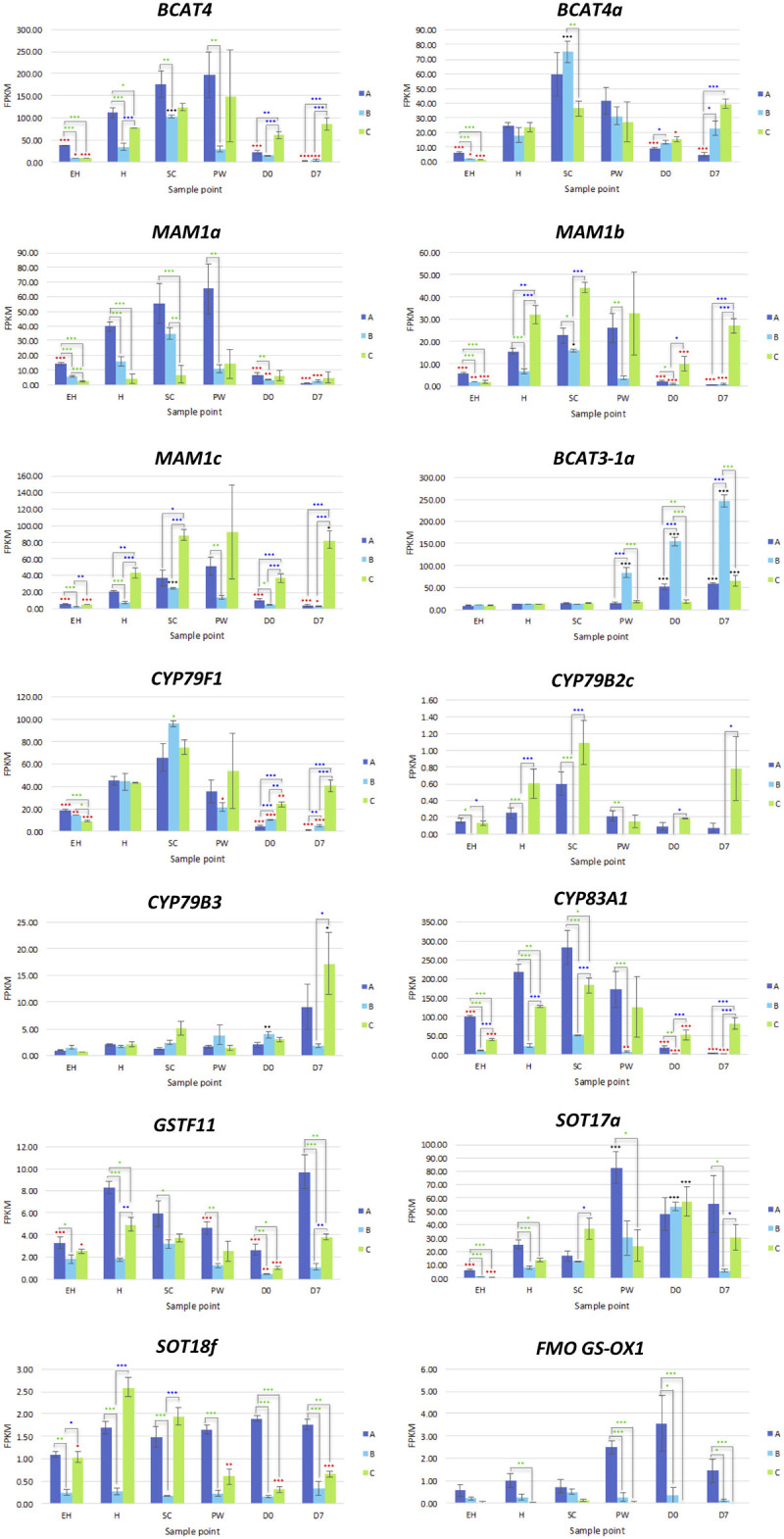
RNAseq expression data (FPKM) for genes involved with glucosinolate biosynthesis in three elite inbred lines of *Eruca sativa* (**A**, dark blue; **B**, light blue; **C**, green). Standard errors of the mean expression values are represented by error bars. Asterisks denote levels of significance of up and down regulation within sample points (between each inbred line) and relative to the point of harvest for each respective sample point: *p ≤ 0.05; **p ≤ 0.01; ***p ≤ 0.001; green, significant up regulation between lines **A**, **B**, and **C**; blue, significant down regulation between lines **A**, **B**, and **C**; black, significant up regulation relative to **H**; red, significant down regulation relative to **H**. **EH**, early harvest; **H**, harvest; **SC**, second harvest; **PW**, pre-wash; **D0**, post-wash; and **D7**, 7-day shelf life.

*MYC2a* and *MYC2c* were highly expressed in line **A**, and had uniform patterns of relative expression. **SC** had the highest expression values for this line, suggesting a general response to mechanical wounding and stress, however this was not significantly different from **H**. The only significant difference for *MYC2c* between **H** and **SC** was in line **B** (a 0.7 log2-fold increase; Figure 7).

*MYB28a* and *MYB28b* display high degrees of differential expression between each rocket line. While **A** has high expression of *MYB28a* in samples **EH**, **H**, **SC**, and **PW**, it is has by comparison lower expression of *MYB28b* compared to **C** (Figure 9). **C** on the other hand has relatively high expression for both of these TFs, and displays significantly higher expression postharvest, up to and including **D7**. Combined with what is known about these TFs in other Brassicaceae species, it is likely that the differences in GSL concentrations observed postharvest are linked to the differential expression of *MYB28a* and *MYB28b* between the respective lines.

Also of note is that expression of MYB28 genes were positively associated with expression of SDI1 gene copies. Previous research has shown that the SDI1 protein binds to MYB28, inactivating expression and reducing GSL biosynthesis (Chan et al., 2019). In *E. sativa* the opposite appears to be true, with significant positive correlations between respective expression of two MYB28 copies and *MYB29* with SDI1 copies (*SDI1a* and *MYB29*, *r* = 0.72; *SDI1b* and *MYB28a*, *r* = 0.507; *SDI1c* and *MYB28c*, *r* = 0.459; Supplementary Data File 1). At **D7**, both **A** and **B** had significantly lower expression levels compared with **H** (a 2.2 and 3.7 log2-fold reduction of *SDI1a*, respectively; and a 3.9 and 3.2 log2-fold significant reduction of *SDI1c*, respectively).

#### Glucosinolate Biosynthesis

Rocket contains two genes encoding BCAT4, and two genes encoding BCAT3; converting 2-oxo acids to homomethionine and dihomomethionine. *BCAT3-1a* displayed no significant variation between lines during growth, but saw significant increases for all (compared to **H**) at **D0** and **D7** (Supplementary Data File 3). The most marked and statistically significant increase was in **B**. It is unclear how this “preference” for BCAT3 activity over BCAT4 is regulated or affects the synthesis pathway, but the relative and respective activity of these genes is associated with GSL content.

**FIGURE 8:**
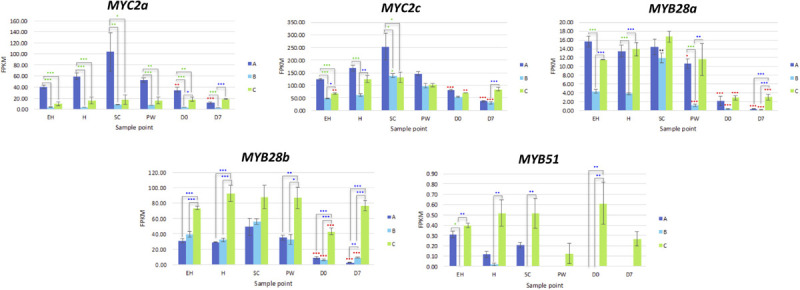
RNAseq expression data (FPKM) for glucosinolate transcription factors in three elite inbred lines of *Eruca sativa* (**A**, dark blue; **B**, light blue; **C**, green). Standard errors of the mean expression values are represented by error bars. Asterisks denote levels of significance of up and down regulation within sample points (between each inbred line) and relative to the point of harvest for each respective sample point: *p ≤ 0.05; **p ≤ 0.01; ***p ≤ 0.001; green, significant up regulation between lines **A**, **B**, and **C**; blue, significant down regulation between lines **A**, **B**, and **C**; black, significant up regulation relative to **H**; red, significant down regulation relative to **H**. **EH**, early harvest; **H**, harvest; **SC**, second harvest; **PW**, pre-wash; **D0**, post-wash; and **D7**, 7-day shelf life.

Only orthologs of MAM1 were identified, with no corresponding MAM2 or MAM3 genes present within the annotation. Each of the three MAM1 copies had differing expression patterns (Figure 8). Line **A** displayed higher relative expression of *MAM1a*, whereas **C** had greater expression for *MAM1b* and *MAM1c*. **B** however maintained low expression for all three of these genes. **A** had reduced expression activity during shelf life, whereas in **C**, levels were significantly higher compared to **H** (Figure 8).

One *CYP79F1* homolog was found in rocket, with no expression found for a corresponding *CYP79F2* gene. The lack of a *CYP79F2* homolog in rocket may be suggestive of a loss of function, and/or redundancy with other enzymes. Of note for *CYP79F1* expression was the significant differences observed between **EH** and **H**, indicating that earlier harvests of rocket leaves may have a reduced ability for GSL biosynthesis compared with later ones and second cuts (**SC**). Expression was significantly greater in **C** during shelf life. In the conversion of aldoximes to nitrile oxides, *CYP83A1* expression was higher in **A** and **C** than **B**, with line **C** having significantly higher expression in shelf life samples (Figure 8).

#### Glucosinolate Hydrolysis

**FIGURE 9:**
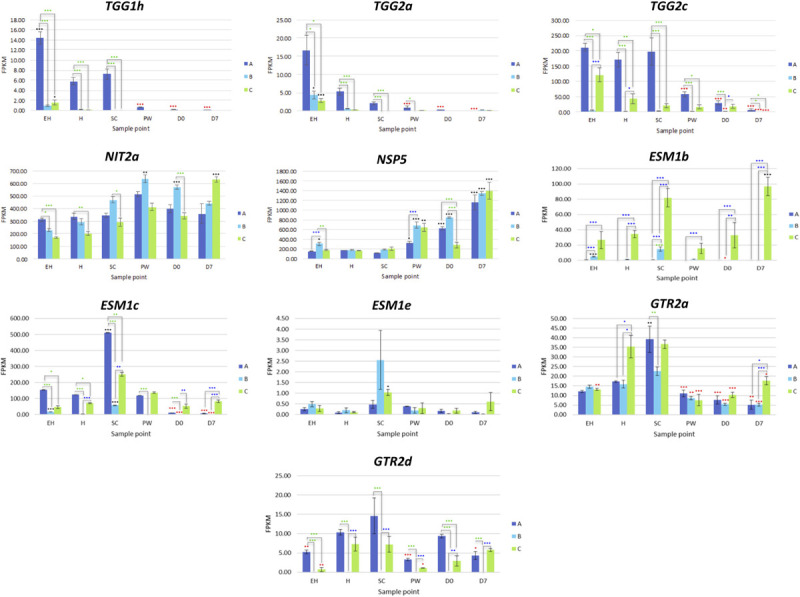
RNAseq expression data (FPKM) for genes involved with glucosinolate hydrolysis and transport in three elite inbred lines of *Eruca sativa* (**A**, dark blue; **B**, light blue; **C**, green). Standard errors of the mean expression values are represented by error bars. Asterisks denote levels of significance of up and down regulation within sample points (between each inbred line) and relative to the point of harvest for each respective sample point: *p ≤ 0.05; **p ≤ 0.01; ***p ≤ 0.001; green, significant up regulation between lines **A**, **B**, and **C**; blue, significant down regulation between lines **A**, **B**, and **C**; black, significant up regulation relative to **H**; red, significant down regulation relative to **H**. **EH**, early harvest; **H**, harvest; **SC**, second harvest; **PW**, pre-wash; **D0**, post-wash; and **D7**, 7-day shelf life.

Eleven TGG1 (myrosinase) and three TGG2 copies (Supplementary Data File 3) were identified within the annotation. Some of these genes appear to have differential expression according to ontogeny and shelf life time point, with some copies expressed at **EH** with none during postharvest (e.g., *TGG1h, TGG2a*, and *TGG2c*; Figure 9). Others however display the inverse of this, with increased relative expression postharvest (*TGG1a*; Supplementary Data File 3). It is known that myrosinases TGG1 and TGG2 are functionally redundant in Arabidopsis, however it has also been noted that their activity and specificity is linked with developmental processes, and may explain some of the high levels of expression observed at **EH**.

An explanation for the lack of nitrile GHPs in rocket may be that the high expression of *NSP5* is inhibited by the five EPITHIOSPECIFIER MODIFIER 1 (ESM1) orthologs found in the rocket genome. These proteins are known to inhibit the action of NSPs and promote ITC formation. Expression was significantly greater in line **C** for *ESM1b* (Figure 11) at all sample points, and fits with the observed pattern of sustained GHP formation postharvest. The lower activities in **A** and **B** did not however correspond to a reciprocal decrease in the relative concentrations of GHPs, and neither were nitrile concentrations at anything above trace levels. Much further work is needed to explain the genetic regulation of GHP formation in rocket and the high expression of *NSP5*.

#### Glucosinolate Transporters

**FIGURE 10:**
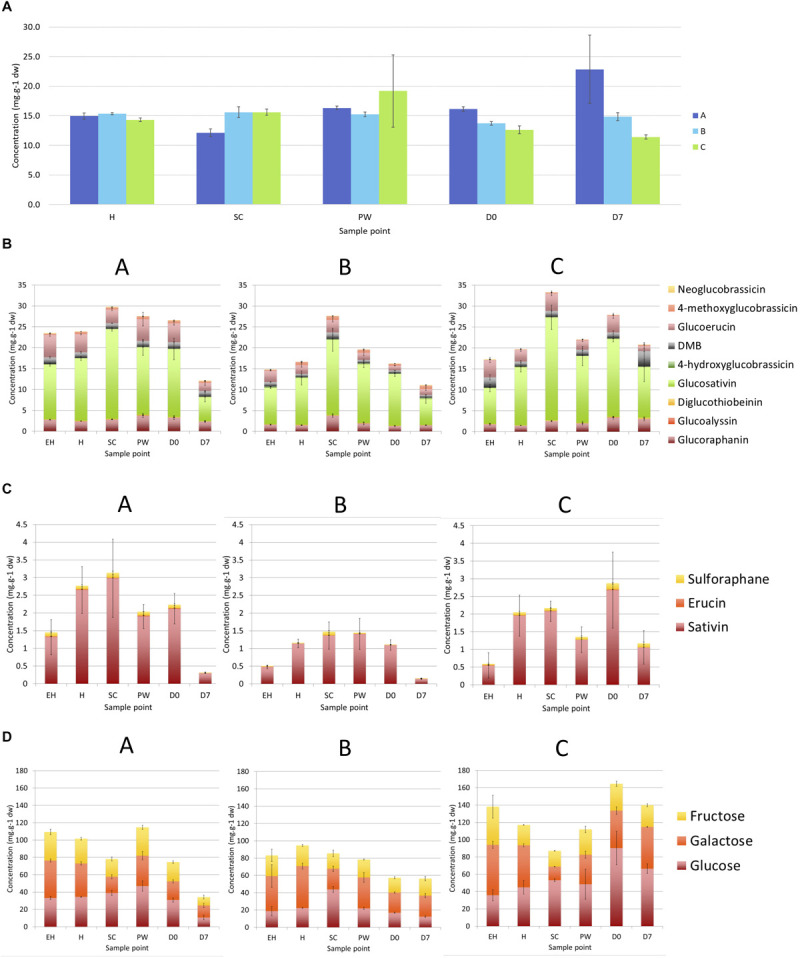
Elemental sulfur **(A)**, glucosinolate **(B)**, glucosinolate hydrolysis product **(C)**, and monosaccharide **(D)** concentrations observed in elite inbred lines of *Eruca sativa* (**A**, **B**, and **C**). Concentrations are expressed as mg.g^– 1^ of dry weight. Error bars represent standard error of the mean of each analyte detected. See insets for compound color coding. For ANOVA and Tukey’s HSD pairwise significance values see Supplementary Data File 1. **EH**, early harvest; **H**, harvest; **SC**, second harvest; **PW**, pre-wash; **D0**, post-wash; and **D7**, 7-day shelf life.

Eight GSL transporter genes were identified in the rocket annotation; four GTR1 and four GTR2 genes. These genes are involved in leaf distribution and long-range phloem GSL distribution, respectively. Expression of *GTR2a* and *GTR2d* were significantly correlated with GSL abundance and GHP formation in the analyzed leaf tissues. Of particular note is that **B** had no expression of *GTR2d* at any of the sample points, indicating that the gene may be non-functional. If this transport system is impaired in **B**, it would explain the significantly lower abundance of GSLs observed in leaves (Figure 10). Coupled with the high expression of glutathione-related genes and similar sulfur content of **B** compared to lines **A** and **C**, the inactivity of this gene copy may have significant effects on leaf sulfur transport, metabolism, and antioxidant response. The lower GSL content in leaves may therefore be compensated by increased glutathione synthesis.

### Sulfur and Phytochemical Composition of *E. sativa*

#### Sulfur Content of *E. sativa*

Total sulfur content for each of the breeding lines is presented in Figure 10A. No significant differences were observed between lines and sample time points (*p* = 0.434). A lack of statistical difference between lines and between time points demonstrates that observed GSL profiles and abundance cannot be inferred from sulfur content of the leaves. As gene expression analysis of sulfur metabolism-related genes has shown, there is a distinct difference between lines **A** and **C** compared with line **B** in the utilization of available sulfur for GSL biosynthesis.

#### Glucosinolate Profiles and Contents of *E. sativa*

For each of the cultivars between the first (**H**) and **SC**, an increase in total GSL concentrations was observed due to elevations of GSV (**A**, a 1.4-fold increase, *p* < 0.0001; **B**, a 1.6-fold increase, *p* < 0.0001; **C**, a 1.8-fold increase, *p* < 0.0001; Figure 10B) and GRA (**B**, a 2.6-fold increase, *p* < 0.0001; **C**, a 1.8-fold increase, *p* < 0.0001; Supplementary Data File 1). Line **C** produced the highest total concentrations of GSLs in **SC** (a 1.7-fold increase; *p* < 0.0001), and line **B** also saw significant elevations compared to **H** (a 1.6-fold increase; *p* < 0.0001).

Line **A** contained the greatest GSL concentrations compared to **B** and **C**, until **D7** where content declined significantly (a 0.6-fold decrease compared to **D0**, *p* < 0.0001; Supplementary Data File 1). **C** by comparison contained high concentrations of GSLs during shelf life, peaking at **D0**, with a non-significant decrease at **D7** (0.3-fold reduction). This line did not demonstrate the same decline in GSLs toward the end of shelf life as in the other two, and displays a propensity for maintaining GSLs for longer into the shelf life period.

#### Glucosinolate Hydrolysis Product Profiles and Contents of *E. sativa*

Glucosinolate hydrolysis product concentrations are presented in Figure 10C (see Supplementary Data File 1 for ANOVA and Tukey’s HSD significances). As with previous studies of rocket (Fechner et al., 2018), three main GHPs were detected: sativin (1,3-thiazepane-2-thione; hydrolysis product of GSV; SAT), erucin (ITC of glucoerucin; GER), and SF. The fluctuations in total GHP concentration mirror those observed for GSLs, however the increases between **H** and **SC** are much less pronounced, with no significant differences between cuts.

As with GSLs, line **B** displayed the lowest concentrations of GHPs, whereas the differences between lines **A** and **C** are less apparent. The trend of reduction of GHPs over shelf life is also visible for lines **A** and **B**, though only significant in **B** (a 0.9-fold reduction, *p* < 0.0001). Concentrations remained higher in line **C** (1.2 mg g^–1^ dw, a 0.6-fold reduction from **D0**).

#### Monosaccharide Profiles and Contents of *E. sativa*

**FIGURE 11:**
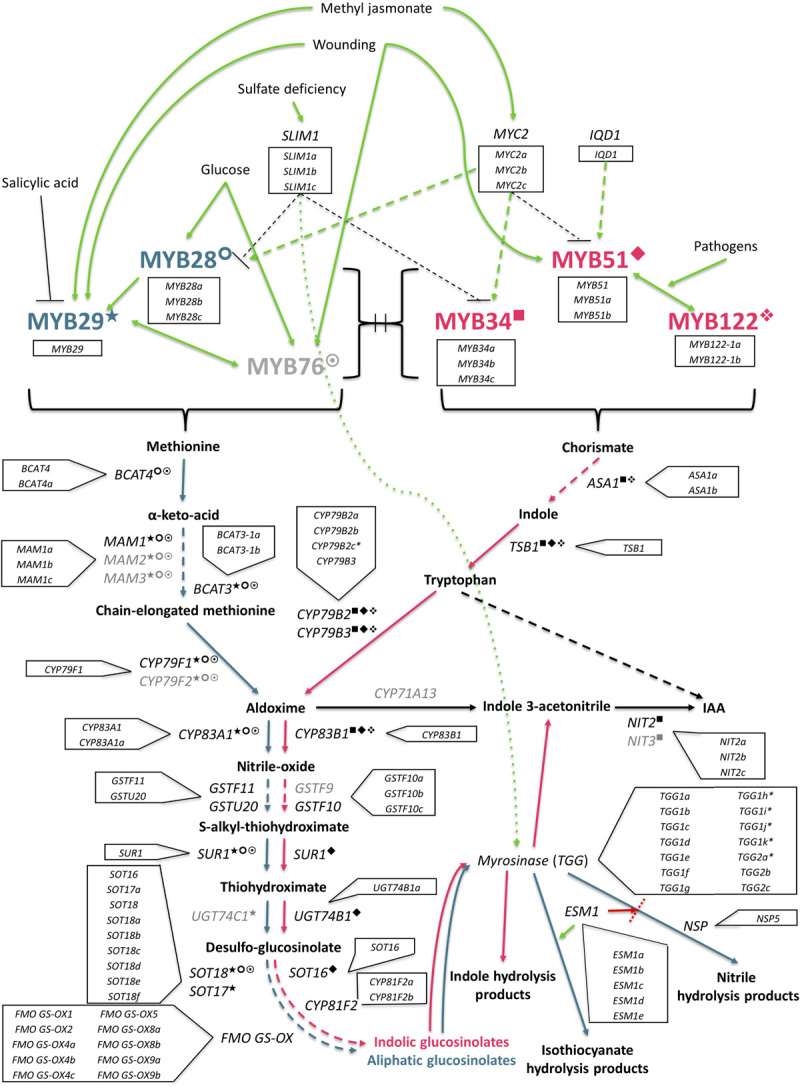
The GSL biosynthesis pathway (adapted from Gigolashvili et al., 2009) is initiated by a complex and interacting network of abiotic and biotic factors. Aliphatic synthesis pathway shown in teal, and the indolic pathway shown in pink, is regulated by R2R3-MYB transcription factors. Known interactions between MYBs and specific genes within each respective pathway are highlighted as follows: ○ MYB28; ★ = MYB29; ☉ = MYB76; ■ = MYB34; ◆ = MYB51; 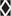 = MYB122. Genes with identified orthologs in the *E. sativa* genome annotation are written in black; those with no identified homologous sequence are written in gray. *SLIM1, sulfur limitation 1*; *IQD1, protein IQ domain 1*; *BCAT, methionine aminotransferase*; *MAM, methylthioalkylmalate synthase*; *CYP, cytochrome P450*; *GST, glutathione-S-transferase*; *SUR1, C-S lyase 1*; *UGT, UDP-glycosyltransferase*; *FMO_*GS–OX*_, flavin-containing monooxygenase*; *ASA1, anthranilate synthase alpha subunit 1*; *TSB1, tryptophan synthase beta chain 1*; IAA, indole-3-acetic acid; *NIT, nitrilase*; *ESM1, epithiospecifier modifier protein 1*; *NSP, nitrile specifier protein*.

Monosaccharides are important in terms of sensory attributes and the masking of bitter and pungent sensory attributes in rocket (Bell et al., 2017a) altering consumer perception and preference. Glucose is also known to influence stress responses and interact with MYB TFs (Gigolashvili et al., 2009; Figure 11). The concentrations of sugars observed in *E. sativa* lines are presented in Figure 10D (see Supplementary Data File 1 for ANOVA and Tukey’s HSD significances).

Unlike previous reports (Bell et al., 2017b) the changes in sugar concentrations in this study were dynamic across each of the respective time points. Both lines **A** and **B** contained low total concentrations compared to line **C**. Line **B** displayed consistent concentrations, with no significant differences observed. **A** showed a similar trend to GSL and GHP concentrations by declining at the end of shelf life (**D7**; a 0.5-fold decrease from **D0**, *p* < 0.0001).

Line **C** is distinct from the others in terms of its sugar profile and the relative differences between sample points. Concentrations increased postharvest (**D0** and **D7**; a 1.4 and 1.2-fold increase relative to **H**, respectively), perhaps owing to a breakdown of stored carbohydrate to facilitate respiration. Line **C** sugar content consists primarily of glucose, whereas **B** tended to have greater concentrations of galactose, and **A** was composed of similar amounts of each monosaccharide.

#### Principal Component Analysis of Sulfur and Glucosinolate Metabolism Genes

**FIGURE 12:**
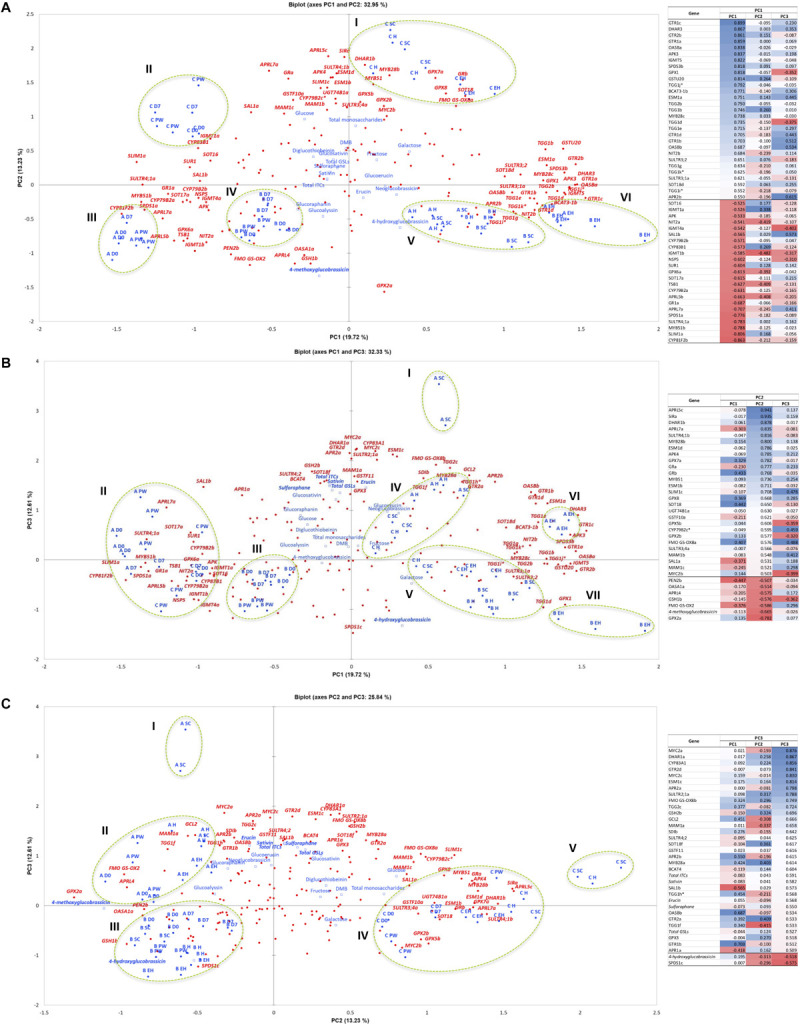
Principal Component Analysis of sulfur assimilation pathway, glucosinolate biosynthesis, and glucosinolate hydrolysis gene expression data (FPKM) for three *Eruca sativa* elite inbred lines (**A**, **B**, and **C**) across ontogenic and postharvest sample points. Biplot **(A)** displays Principal Components (PCs) 1 and 2, which represent 33% of variation within the data. Biplot **(B)** displays PC1 and PC3, explaining 32.3% of variation within the data; and **(C)** displays PC2 and PC3, explaining 25.8% of the variability. The PCA plots presented are the results of Varimax rotation. Each biplot is accompanied by a factor loadings table sorted according to PC1 **(A)**, PC2 **(B)**, and PC3 **(C)**; italics denotes a supplementary variable, and asterisks denotes a putative novel gene within the reference annotation. Blue coloration denotes high factor loading scores, red denotes low. Only genes with loading values >0.5 were included, and each is represented within the biplots in red (bold italics). Red circles represent individual genes included in the analysis (*n* = 177). Blue circles represent sample point variables and have accompanying labels (blue bold). Blue squares denote phytochemical data regressed onto the PCA as supplementary variables. Bold data labels indicate phytochemical components with >0.5 factor loadings scores. Green dotted ellipses denote clusters of variables and are numbered using Roman numerals, which are quoted within the text. **EH**, early harvest; **H**, harvest; **SC**, second harvest; **PW**, pre-wash; **D0**, post-wash; and **D7**, 7-day shelf life.

Hereafter, only correlations significant at the *p* < 0.001 level are presented and discussed in relation to the PCA. *SULTR4;1a* was significantly correlated with GRA concentrations (*r* = 0.577), which is associated with shelf life samples for lines **A** and **C** (Figure 12B, cluster **II**). Figures 12A,B show a distinct separation between ontogenic and shelf life samples along PC1. The increased expression of sulfur transport genes such as this postharvest may provide some explanation as to why GSL concentrations increase in the initial stages shelf life (**PW**), as S may be re-mobilized to facilitate biosynthesis. Efficient transport and storage of sulfur pre-harvest may also facilitate better retention and decreased degradation of GSLs postharvest. This can be seen in Figure 12B (**V**) where *SULTR3;1a* and *SULTR3;2* are associated with pre-harvest expression.

Sulforaphane and SAT concentrations were significantly correlated with *APR2a* gene expression (Figure 12C **I** and **II**; *r* = 0.58, SF; *r* = 0.464, SAT) and associated in particular with **A** ontogenic samples and **PW**. *APR2* is known to contribute to sulfur accumulation and homeostasis, as well as facilitating cysteine synthesis, and is associated with increased myrosinase activity and GSL recycling. Line **A** (on average) contained the highest ontogenic concentrations of GRA, SF, GSV, and SAT (Figures 10B,C); this is supported by a significant correlation and association with GSL-related TFs *MYB28a* (*r* = 0.486, SF; *r* = 0.53, SAT), *MYC2a* (*r* = 0.596, SF; *r* = 0.626, SAT) and *MYC2c* (*r* = 0.584, SF; *r* = 0.583, GSV; *r* = 0.634, SAT; Figure 12C **I** and **II**), as well as a drought tolerance-related gene *SAL1b* (*r* = 0.595, GRA; *r* = 0.547, SF; *r* = 0.499, SAT; Figures 12A **II**, **III** and **IV**, **12C II**). **A** was also associated with increased activity of *MAM1a* (Figure 12C **II**), facilitating greater GRA biosynthesis through chain elongation. It may be that lines **A** and **C** have increased relative GRA concentrations at **EH** and **H**, but preferentially express *MYB28c* and *MYB28b*, respectively. It is unknown if the function of each MYB28 TF is redundant in rocket, but these data would suggest that there is some clear overlap of function, though the expression of *MYB28b* in particular is associated with increased GSL biosynthesis postharvest (Figure 8).

The lower relative expression in line **B** for many of these genes is consistent with its lower GSL and hydrolysis product concentrations, irrespective of sample point. GRA/SF, GSV/SAT, and GER concentrations were significantly and negatively correlated with *SPERMIDINE SYNTHASE 1c* (*SPDS1c*; *r* = −0.622, GRA; *r* = −0.614, SF; *r* = −0.6, GSV; *r* = −0.454, SAT; *r* = −0.604, GER), which had a high degree of co-separation in all **B** samples (Figure 12C **III**). This association may be related to increased primary S metabolism and reduced partitioning of methionine for secondary S metabolites (Figure 1).

## Discussion

### The Complexity of the *E. sativa* Genome and Future Novel Gene Discovery

The presented reference genome and annotation for *E. sativa* shows a huge amount of complexity. The transcriptomic evidence presented here also illustrates just how variable traits and expression can be between breeding lines under controlled environmental conditions. In the three inbred lines tested, global differential expression of genes was highly variable, and suggests mechanisms present in commercial rocket that underlie differences in postharvest quality and shelf life performance. This is exemplified in line **C**, which displayed differential patterns of expression, both during growth stages and postharvest (Supplementary Data File 3). A literature search suggests some studies treat cultivars of *E. sativa* the same, without regard for potential differences in phytochemistry or postharvest quality (Jin et al., 2009; Selma et al., 2010), and that produce is genetically uniform. This study demonstrates wide variation between genotypes, and there is significant potential for further crop improvement for enhanced shelf life nutritional quality. Development of these data in *Eruca* is a major step forward for a crop once considered to be niche, and which now joins a growing list of minor crops in the genomic era. While this study has highlighted the orthologous genes that are likely to be involved with sulfur assimilation, GSL accumulation, and postharvest stress response, much further research will be needed to unpick novel gene functions and interactions.

### *Eruca sativa* Has a Distinct and Complex Glucosinolate Pathway

Evolutionary divergence between *E. sativa* and other Brassicales species has led to a unique GSL synthesis pathway, displaying extensive gene duplication. Aside from the duplications of MYB28 found in salad rocket, one TF prominent in GSL biosynthesis has no orthologous sequence or expression in the tested rocket lines: MYB76. Similarly, other genes, such as MAM2, MAM3, CYP79F2, CYP71A13, GSTF9, UGT74C1, and NIT3, are all absent from the reported rocket genome annotation. While this may be amended with future annotation iterations and sequence improvements, it is conceivable that these genes may have been lost over the course of evolutionary time and divergence with *A. thaliana*.

It is not clear what the function(s) of gene copies and paralogues may be in *Eruca*. It may be the result of segmental duplications within the genome, such as those observed in the *Brassica* A genome (Jiang et al., 2011), and future, more detailed studies of the *Eruca* genome structure may reveal the nature and number of any such events. For example, *B. rapa* and *B. oleracea* contain two copies of SOT18 (Liu et al., 2014), whereas in *E. sativa* we report seven. *B. rapa* has two copies of FMO_*GS–OX*_ genes, and salad rocket has at least ten. The related *Diplotaxis tenuifolia* (“wild” rocket) transcriptome has been reported to contain three copies of MYB28 (Cavaiuolo et al., 2017), and is consistent with the hypothesis that duplication occurred after *Eruca* and *Diplotaxis* diverged with a common ancestor in the *B. oleracea* lineage.

One example of recent novel gene discovery outside of *Arabidopsis* and *Brassica* species GSL synthesis pathways is *GLUCORAPHASATIN SYNTHASE 1* (*GRS1*) in radish; which is thought to have evolved from a mutation in a 2-oxoglutarate-dependent dioxygenase (2OGD; Kakizaki et al., 2017). Similar mutations and modification of genes have likely occurred in *E. sativa* and led to the evolution of GSV, GRL, DGTB, and DMB. Future work will elucidate the genes responsible for synthesis of these GSLs. The development of the genomic and transcriptomic resources in this study are an important first step in achieving this.

### Genes in Sulfate and Glucosinolate Pathways Are Strongly Correlated With Glucosinolate Biosynthesis

Principal Component Analysis highlighted several genes that are significantly correlated with the abundance of GSLs in *E. sativa*. In terms of sulfur assimilation, the expression of genes *SULTR4;1b* and *APR2a* appear to be strongly associated with both aliphatic and indolic GSL biosynthesis. *SULTR4;1* facilitates transport of sulfate from cell vacuoles into the cytoplasm, and has been previously linked with the activity of MYB28 and MYB29 (Sønderby et al., 2010), which is supported by this study. Likewise, co-expression analysis found that *MYB28b* and *SiRa* belong to the same gene module (**M6**); suggesting a transcriptional relationship between aliphatic GSL biosynthesis and primary sulfur metabolism. It may be that expression of *MYB28b* expedites the synthesis of GSLs by facilitating greater availability of sulfate. This is in turn linked with the activity of *APR2*, which is known to be responsible for regulating sulfur homeostasis (Kopriva et al., 2015). This gene has also been associated with increased GSL recycling and myrosinase activity (Maruyama-Nakashita et al., 2003). It is likely that the transport of sulfate and its management in terms of recycling is pivotal for GSL abundance and flux in rocket at any given time.

### Postharvest Maintenance of Glucosinolate Content Is Related to Senescence and Oxidative Stress

As shown in Figure 10A, the content of sulfur between the three tested breeding lines was not significantly different. In light of the observed differences in gene expression and GSL accumulations, we theorize that primary and secondary sulfur metabolism pathways “compete” for assimilated environmental sulfur. As content was not significantly different postharvest (**PW**, **D0**, and **D7**) compared to pre-harvest first cut (**H**) in any of the breeding lines, the degree of remobilization and ability to synthesize/recycle GSLs is under strict genetic control. The evolutionary advantages of this are unclear, but as shown in Figure 10B, the amount of total sulfur assimilated during growth is not reflected in the postharvest concentrations of GSLs. Line **B** exemplifies this: it contains statistically no more or less sulfur than lines **A** or **C**, yet synthesizes far fewer GSLs and any given time point.

The natural strategy of the leaf is to remobilize sulfur around parts of the plant as required, such as in times of deficiency. The transcriptome of severed leaves in the postharvest context is an evolutionary dead end, and not subject to natural selection. As such, the differences we have observed can be attributed to different strategies for dealing with unexpected physiological stress, nutrient deficiencies, or as part of senescence responses. This is exemplified by the high relative expression of **M8** (Figure 6) in line **B**, where *SEN1* and *OXS3* are present; suggesting a deficiency in its ability to cope with oxidative stress compared to lines **A** and **C**.

### Co-expression Patterns Reveal Possible Links Between Indole Glucosinolate Biosynthesis, Hydrolysis and Catabolism

The identified modules of expression contained genes involved with or linked to GSL biosynthesis and hydrolysis. Notably, module **M1** contains a number of indole GSL biosynthesis genes and myrosinases. In other Brassicales, indolic GSL biosynthesis is being increasingly linked with auxin and camalexin biosynthesis in related species, via shared reactions with indole-3-acetaldoxime (IAOx; Malka and Cheng, 2017). The high activity of such indole GSL-related genes in rocket suggests that indole GSLs have a high turnover *in planta*, as concentrations are typically low in rocket (Figure 10B). This is also supported by the high expression of genes such as *NIT2a* (Figure 9), which codes for nitrilase involved in the metabolism of indole-3-acetic acid (IAA). The co-expression of several myrosinases (TGG1s and TGG6s’) within **M1** suggests that the hydrolysis of indole GSLs is intrinsically tied to these catabolic processes, and may therefore explain why indolic GSLs are found in such relatively low concentrations in rocket compared to other species (such as *B. oleracea*). The diversity of myrosinase genes identified within the rocket genome and the association of specific copies in **M1** also implies that these may have evolved specific paralogous functions within the indole-GSL pathway. These data therefore provide new insights into the role of indolic GSLs in non-model species, and numerous avenues for future study.

### The Number of Identified Myrosinase Gene Copies Is Indicative of Specialized Functionalities

Perhaps of most interest and significance in this study is the high copy numbers of myrosinase genes (TGGs) present in the *Eruca* annotation. Both *Arabidopsis* and *B. rapa* have four myrosinase gene copies of TGG1 and TGG2, and *B. oleracea* has six (Liu et al., 2014). Our data indicate that *Eruca* has at least 14 copies, as well as two copies encoding PEN2 myrosinase. There has evidently been a massive diversification and duplication of these enzymes in rocket, but it has yet to be established if this is reflected in functionality and spatial expression. The high number of identified TGG1, TGG2, and (poorly characterized) TGG6 genes in *Eruca* also suggests diversified function, as paralogous gene duplications relieve the evolutionary pressure upon orthologous genes; thereby allowing for redundancy with the original function, and subsequently diversification of function over the course of evolutionary time (Selzer et al., 2018). The presented annotation therefore provides new information regarding myrosinase and PEN2 variability.

Such duplications demonstrate the importance of the pathway, offering resilience against random mutations and/or loss of function. Divergence indicates the roles of GSLs (and their associated downstream and upstream metabolites) are under evolutionary pressure to adapt to environmental conditions; perhaps as a means of deterring feeding insects or protecting against infections when cells are damaged. The duplication of myrosinase genes may also be linked to the unusual GSL profile of salad rocket, which contains several compounds not found outside of the *Eruca* and *Diplotaxis* genera. The mechanisms behind hydrolysis of compounds such as GSV and DMB are presently unknown, and the hydrolysis products of GRL and DGTB have not yet been identified. Similarly, the co-expression of TGG1 and TGG6 myrosinases with indolic GSL biosynthesis genes (Supplementary Data File 2) suggests specific functionality and involvement with catabolic processes therein. Through co-expression analysis we have identified four candidate myrosinases in gene module **M1** for future investigation.

## Conclusion

*Eruca sativa* is a promising crop for future improvement, having numerous nutritional and sensory quality traits of benefit and interest to the consumer. We have produced the first reference genome sequence and annotation for the species that will aid in these efforts. The transcriptomic information associated with different harvest and shelf life time points indicates that there are complex mechanisms governing the nutritional quality of rocket leaves, that links sulfur metabolism, GSL biosynthesis, senescence, and oxidative stress responses. Through co-expression analysis we have identified multiple genes for future studies to target. These data will also assist in understanding how the unique GSL compounds found in *E. sativa* are synthesized, and what functions they have within the plant.

## Data Availability Statement

The raw reads, full reference genome sequence, and annotation can be found in the European Nucleotide Archive (Sample accession no. SAMEA13191861; Study ID: PRJEB50993; Sample IDs: ERX8524212, ERX8524213, ERX8524214, ERX8524215).

## Author Contributions

LB and CW conceived and designed the experiment and analyses. RT and SK produced the breeding line seed material for genome sequencing and the RNAseq experiment. LB grew plants in controlled environment, performed RNA extractions and quality control, qRT-PCR validation, glucosinolate analysis by LC-MS, hydrolysis product analysis by GC-MS, performed ANOVAs, Pearson’s correlation analyses, co-expression module analysis, and Principal Component Analyses of phytochemical and gene expression data. MP performed sugar analysis by HPLC. MC performed sulfur content analysis by ICP-OES. LB wrote the manuscript, with contributions from MC, RT, SK, LM, and CW. LB, LM, and CW obtained the funding. All authors contributed to the article and approved the submitted version.

## Conflict of Interest

RT and SK are employed by the company Elsoms Seeds Ltd. The remaining authors declare that the research was conducted in the absence of any commercial or financial relationships that could be construed as a potential conflict of interest.

## Footnotes

1
http://broadinstitute.github.io/picard/

2
http://www.repeatmasker.org/

3
http://www.repeatmasker.org/RepeatModeler.html

4
http://homepage.mac.com/iankorf/

5
http://bioinfo.ut.ee/primer3/

## Supplementary Material

Supplementary Figure 1 RNAseq experimental design and sampling diagram. Three elite inbred lines of *Eruca sativa* were grown under controlled environment conditions and sampled at each of the six time points indicated (in triplicate). **EH**, early harvest; **H**, harvest; **SC**, second harvest; **PW**, pre-wash; **D0**, post-wash; and **D7**, 7-day shelf life.

Supplementary Figure 2 Pearson correlation matrix of RNAseq biological sample replicate gene expression values. Replicates of each sample showed a high degree of correlation (*r*^2^ = >0.884) indicating robust reproducibility of gene expression between the individual plants tested at each respective sample point.

Supplementary Figure 3 qRT-PCR (green) vs. RNAseq (orange) gene expression of ten randomly selected glucosinolate biosynthesis and hydrolysis-related genes. Data are expressed as the normalized log2-fold change in expression relative to the reference gene *ACT11*. ANOVA revealed no significant difference between the two data sets. **EH**, early harvest; **H**, harvest; **SC**, second harvest; **PW**, pre-wash; **D0**, post-wash; and **D7**, 7-day shelf life.

Supplementary Table 1 Genome sequencing pooled DNA sample quality control data for three *Eruca sativa* elite inbred lines.

Supplementary Table 2 RNAseq sample quality control data.

Supplementary Table 3 qRT-PCR primers and efficiencies.

Supplementary Table 4 Numbers of genes with homology or functional assignment.

Supplementary Data File 1 Analysis of Variance outputs with Tukey’s HSD pairwise comparisons between sample points and each respective rocket breeding line: Tab 1 – glucosinolate analysis; Tab 2 – GHP analysis; Tab 3 – sugar analysis. Highlighted values are significant at the following levels: *p* = < 0.05 (yellow), *p* = < 0.01 (orange), and *p* = < 0.001 (green). Tab 4 contains a Pearson’s correlation analysis matrix for sulfur and glucosinolate-related gene expression values and phytochemical observations. Values in bold are significant correlations at the *p* = < 0.001 threshold.

Supplementary Data File 2 List of genes identified by CEMiTool (Co-Expression Module Analysis) and the associated enrichment analysis statistics for each *Eruca sativa* genotype. Contains RNAseq read counts, log2-fold changes, *p*-values, and *q*-values (padj) for all genes within modules M1 to M8 for each of the three rocket lines and the respective sample points. Significant up/down regulation is denoted by green/red highlighting, respectively. KEGG annotation numbers and UniProt gene descriptions for orthologous genes in *A. thaliana* are provided.

Supplementary Data File 3 RNAseq read counts, log2-fold changes, *p*-values, and *q*-values (padj) for sulfur metabolism, glucosinolate biosynthesis, hydrolysis, and transport genes for each of the three rocket lines and the respective sample points. Significant up/down regulation is denoted by green/red highlighting, respectively. KEGG annotation numbers and UniProt gene descriptions for orthologous genes in *A. thaliana* are provided.
