# Corrigendum: The *Eruca sativa* genome and transcriptome: A targeted analysis of sulfur metabolism and glucosinolate biosynthesis pre and postharvest

**DOI:** 10.3389/fpls.2022.1026101

**Published:** 2022-09-23

**Authors:** Luke Bell, Martin Chadwick, Manik Puranik, Richard Tudor, Lisa Methven, Sue Kennedy, Carol Wagstaff

**Affiliations:** ^1^ School of Agriculture, Policy and Development, University of Reading, Reading, United Kingdom; ^2^ School of Chemistry Food and Pharmacy, University of Reading, Reading, United Kingdom; ^3^ Elsoms Seeds Ltd., Spalding, United Kingdom

**Keywords:** rocket (*Eruca sativa and Diplotaxis tenuifolia*), isothiocyanate, postharvest, sulfur assimilation, glucosinolate transport, transcription factor, MYB28, SLIM1

In the published article, there was an error in the Data Availability statement. The European Nucleotide Archive Study ID was displayed as “PRJEB34051”, with Accession no. “GCA_902460325” and Sample ID: “ERS3673677”. The correct Data Availability statement appears below.

## Data availability statement

The raw reads, full reference genome sequence, and annotation can be found in the European Nucleotide Archive (Sample accession no. SAMEA13191861; Study ID: PRJEB50993; Sample IDs: ERX8524212, ERX8524213, ERX8524214, ERX8524215).

The authors apologize for this error and state that this does not change the scientific conclusions of the article in any way. The original article has been updated.

## Publisher’s note

All claims expressed in this article are solely those of the authors and do not necessarily represent those of their affiliated organizations, or those of the publisher, the editors and the reviewers. Any product that may be evaluated in this article, or claim that may be made by its manufacturer, is not guaranteed or endorsed by the publisher.

