# Supplementary figures and images for: The Eruca sativa Genome and Transcriptome: A Targeted Analysis of Sulfur Metabolism and Glucosinolate Biosynthesis Pre and Postharvest
Source: Front Plant Sci. 2020 Oct 27;11:525102. doi: 10.3389/fpls.2020.525102 (PMC7652772; doi:10.3389/fpls.2020.525102)

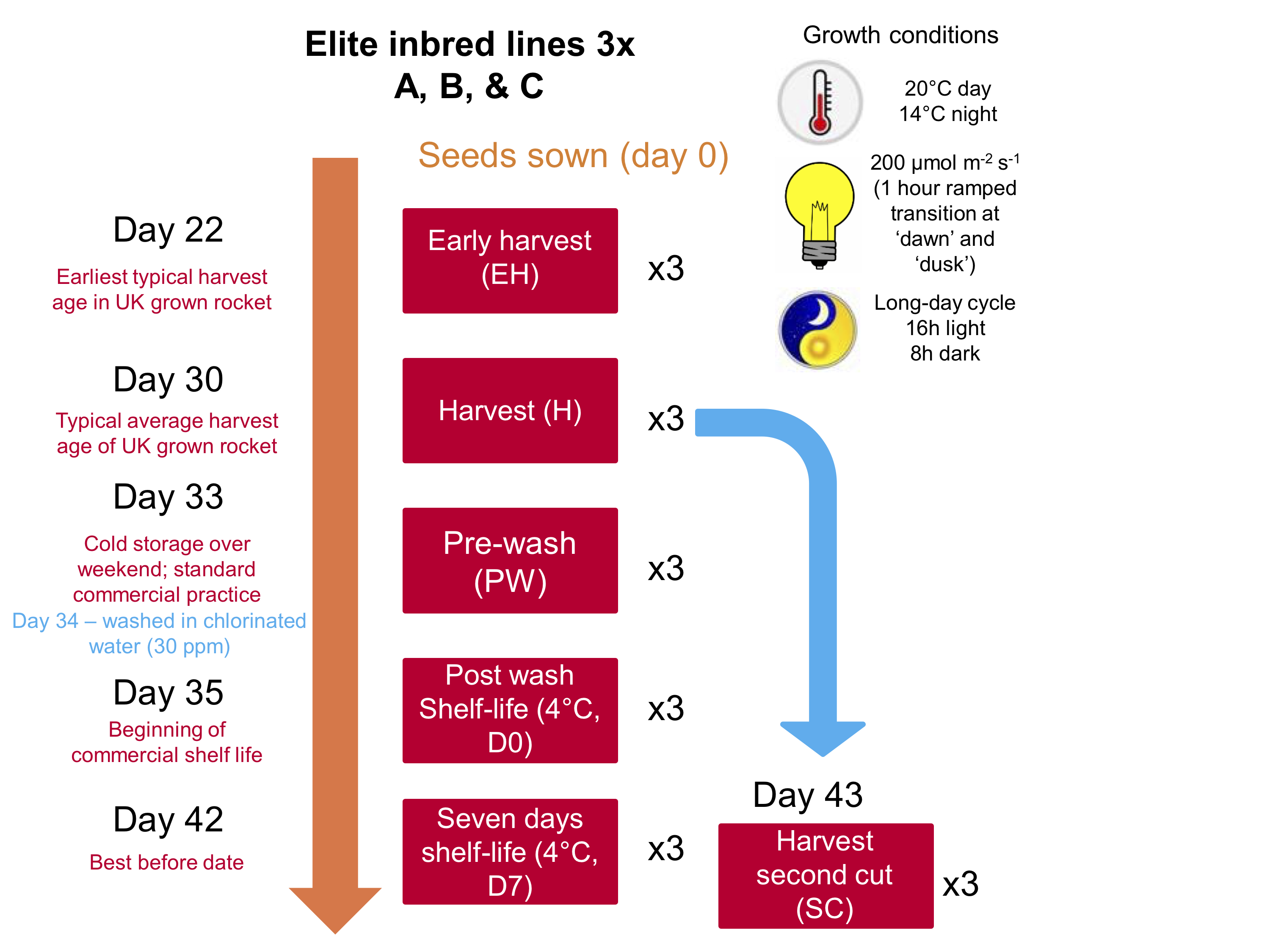

Supplement: Supplementary Figure 1 — RNAseq experimental design and sampling diagram. Three elite inbred lines of Eruca sativa were grown under controlled environment conditions and sampled at each of the six time points indicated (in triplicate). EH, early harvest; H, harvest; SC, second harvest; PW, pre-wash; D0, post-wash; and D7, 7-day shelf life. [file Image_1.TIFF]

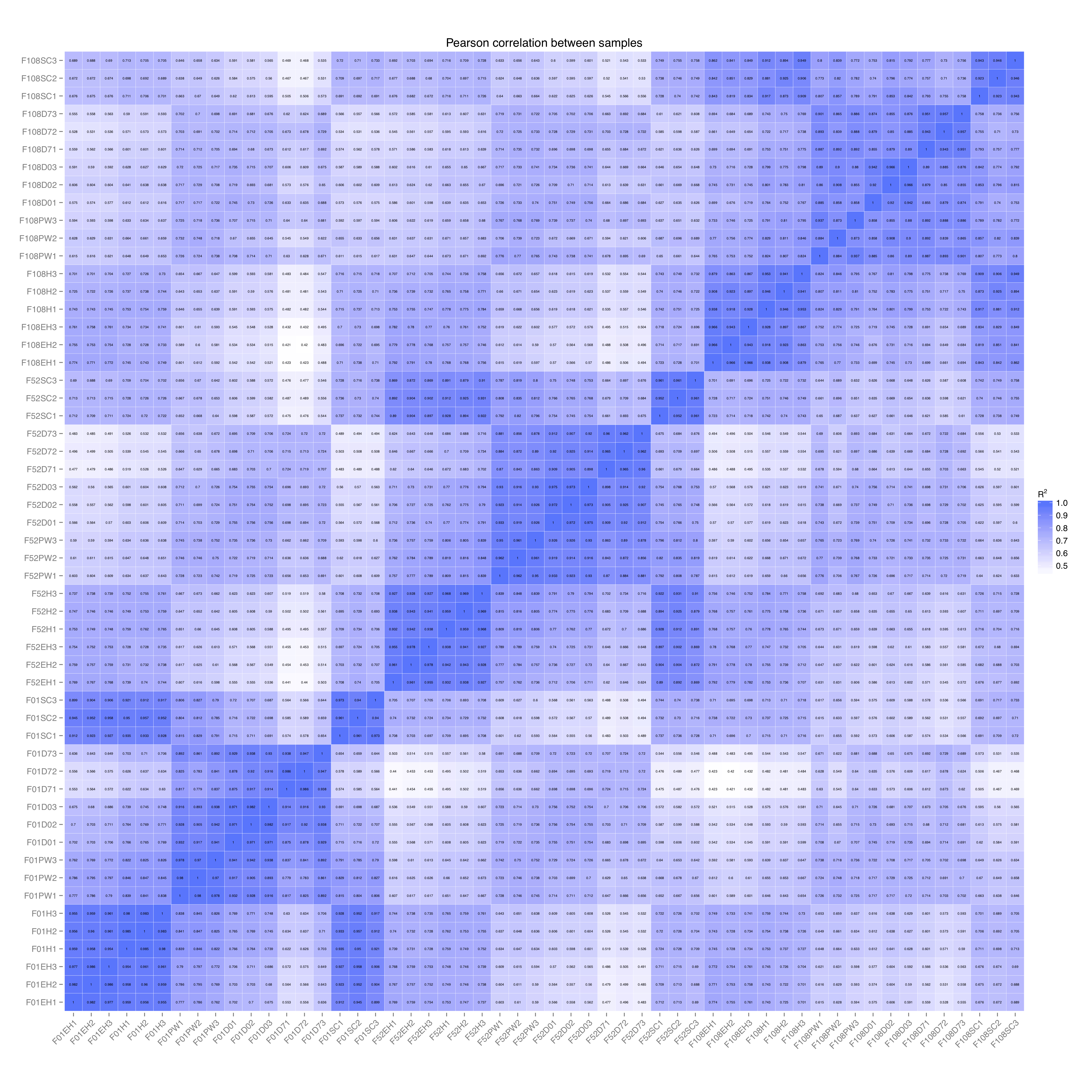

Supplement: Supplementary Figure 2 — Pearson correlation matrix of RNAseq biological sample replicate gene expression values. Replicates of each sample showed a high degree of correlation (r2 = >0.884) indicating robust reproducibility of gene expression between the individual plants tested at each respective sample point. [file Image_2.JPEG]

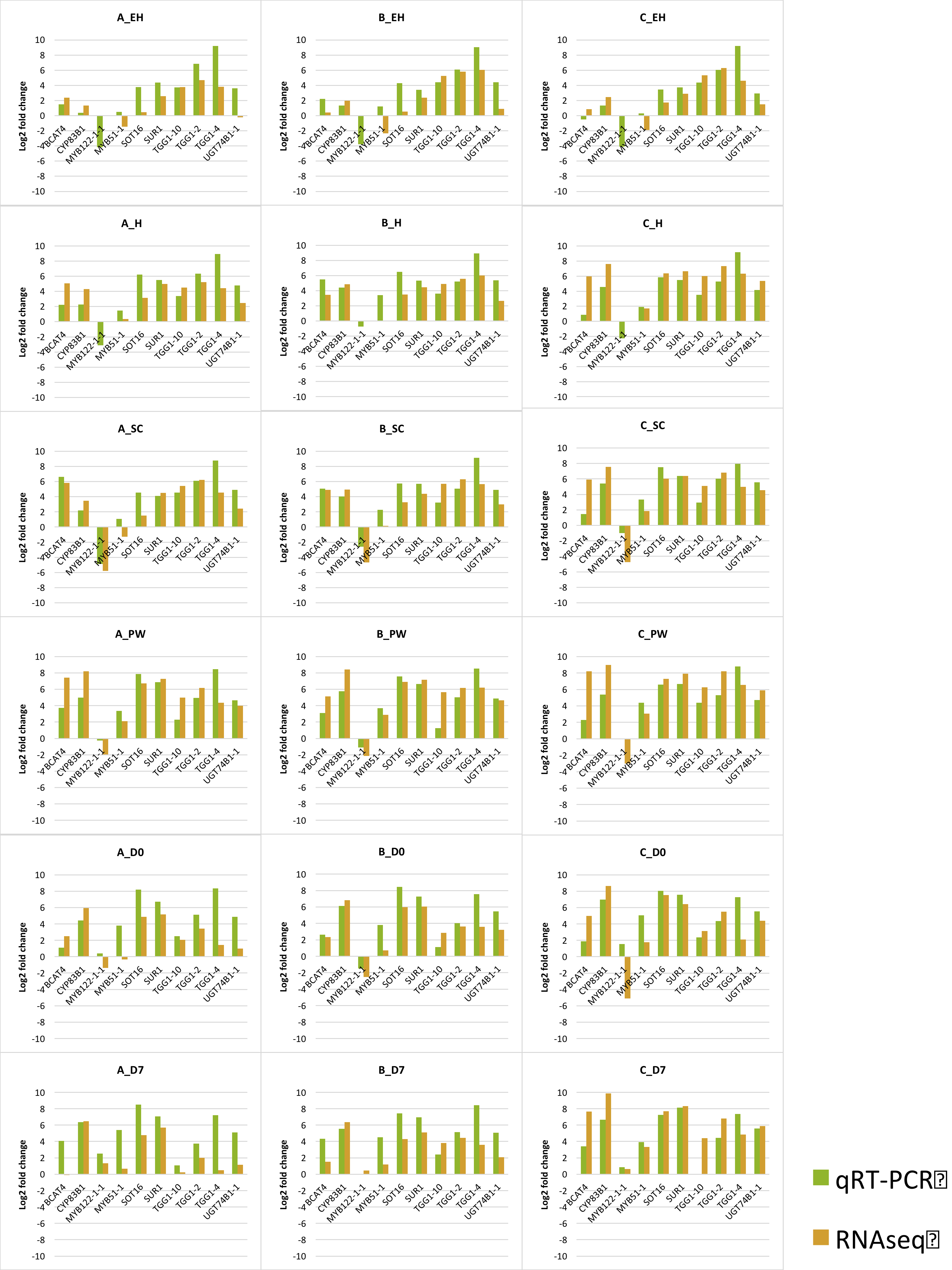

Supplement: Supplementary Figure 3 — qRT-PCR (green) vs. RNAseq (orange) gene expression of ten randomly selected glucosinolate biosynthesis and hydrolysis-related genes. Data are expressed as the normalized log2-fold change in expression relative to the reference gene ACT11. ANOVA revealed no significant difference between the two data sets. EH, early harvest; H, harvest; SC, second harvest; PW, pre-wash; D0, post-wash; and D7, 7-day shelf life. [file Image_3.JPEG]
