# Supplementary material for: The Eruca sativa Genome and Transcriptome: A Targeted Analysis of Sulfur Metabolism and Glucosinolate Biosynthesis Pre and Postharvest
Source: Front Plant Sci. 2020 Oct 27;11:525102. doi: 10.3389/fpls.2020.525102 (PMC7652772; doi:10.3389/fpls.2020.525102)
Supplement: Supplementary Table 1 — Genome sequencing pooled DNA sample quality control data for three Eruca sativa elite inbred lines. [file Table_1.DOCX]

| **Table S1.** Genome sequencing pooled DNA sample quality control data for three *Eruca sativa* elite inbred lines | | | | | | | |
| --- | --- | --- | --- | --- | --- | --- | --- |
| **Sample** | **Concentration (ng µL^-1^)** | **Sample volume (µL)** | **Quantification methods** | **260/280 ratio** | **260/230 ratio** | **Buffer used** | **Extraction kit used** |
| A | 23.2 | 120 | Nanodrop + Qubit | 2.31 | 1.04 | 10 mM Tris, pH 8.0 | E.Z.N.A. Plant DNA DS Mini Kit |
| B | 29.3 | 120 |  | 2.04 | 2.14 |  |  |
| C | 43.2 | 120 |  | 2.00 | 2.31 |  |  |
