# Supplementary material for: The Eruca sativa Genome and Transcriptome: A Targeted Analysis of Sulfur Metabolism and Glucosinolate Biosynthesis Pre and Postharvest
Source: Front Plant Sci. 2020 Oct 27;11:525102. doi: 10.3389/fpls.2020.525102 (PMC7652772; doi:10.3389/fpls.2020.525102)
Supplement: Supplementary Table 2 — RNAseq sample quality control data. [file Table_2.DOCX]

| **Table S2.** RNAseq sample quality control data | | | | |  |  |  |  |
| --- | --- | --- | --- | --- | --- | --- | --- | --- |
| Line | Time point | Replicate | Conc. (ng µL^-1^) | Amount (µg) | | 260/280 | 260/230 | RNA Integrity No. |
| A | EH | 1 | 598 | 14.35 | | 2.14 | 2.25 | 5.9 |
| A | EH | 2 | 648 | 14.26 | | 2.12 | 1.84 | 4.7 |
| A | EH | 3 | 498 | 11.95 | | 2.15 | 1.98 | 3.8 |
| A | H | 1 | 582 | 14.55 | | 2.14 | 2.27 | 5.6 |
| A | H | 2 | 405 | 10.13 | | 2.13 | 2.29 | 4.8 |
| A | H | 3 | 540 | 13.50 | | 2.16 | 2.35 | 5.2 |
| A | PW | 1 | 405 | 9.72 | | 2.10 | 1.71 | 4.9 |
| A | PW | 2 | 368 | 8.83 | | 2.12 | 2.17 | 5.1 |
| A | PW | 3 | 386 | 8.88 | | 2.03 | 1.87 | 5.1 |
| A | D0 | 1 | 368 | 8.83 | | 2.09 | 2.19 | 6.3 |
| A | D0 | 2 | 628 | 14.44 | | 2.14 | 2.23 | 5.1 |
| A | D0 | 3 | 400 | 10.00 | | 2.02 | 2.22 | 5.2 |
| A | D7 | 1 | 314 | 7.54 | | 2.12 | 2.09 | 3.7 |
| A | D7 | 2 | 282 | 6.77 | | 2.17 | 1.88 | 3.5 |
| A | D7 | 3 | 440 | 10.56 | | 2.14 | 2.18 | 4.3 |
| A | SC | 1 | 112 | 2.46 | | 2.00 | 2.00 | 4.6 |
| A | SC | 2 | 1130 | 27.12 | | 1.99 | 1.93 | 3.8 |
| A | SC | 3 | 214 | 4.92 | | 2.18 | 2.14 | 7.3 |
| B | EH | 1 | 930 | 22.32 | | 2.15 | 2.10 | 5.5 |
| B | EH | 2 | 998 | 23.95 | | 2.15 | 2.20 | 5.6 |
| B | EH | 3 | 1114 | 26.74 | | 2.06 | 1.75 | 6.2 |
| B | H | 1 | 534 | 12.81 | | 2.14 | 2.30 | 5.4 |
| B | H | 2 | 1060 | 25.44 | | 2.09 | 2.14 | 5.4 |
| B | H | 3 | 812 | 19.49 | | 2.14 | 2.32 | 5.4 |
| B | PW | 1 | 1020 | 24.48 | | 2.14 | 2.33 | 5.6 |
| B | PW | 2 | 974 | 23.38 | | 2.15 | 2.39 | 5.2 |
| B | PW | 3 | 770 | 18.48 | | 2.13 | 2.39 | 5.8 |
| B | D0 | 1 | 679 | 16.30 | | 2.14 | 2.40 | 5.8 |
| B | D0 | 2 | 920 | 22.08 | | 2.17 | 2.42 | 6.0 |
| B | D0 | 3 | 749 | 17.98 | | 2.12 | 2.20 | 5.1 |
| B | D7 | 1 | 791 | 18.98 | | 2.14 | 2.40 | 5.2 |
| B | D7 | 2 | 602 | 14.45 | | 2.14 | 2.37 | 5.5 |
| B | D7 | 3 | 640 | 15.36 | | 2.11 | 2.34 | 5.7 |
| B | SC | 1 | 166 | 3.82 | | 2.13 | 2.02 | 5.0 |
| B | SC | 2 | 276 | 6.62 | | 2.10 | 2.31 | 5.3 |
| B | SC | 3 | 116 | 2.67 | | 2.00 | 2.00 | 5.2 |
| C | EH | 1 | 750 | 18.00 | | 2.13 | 2.39 | 6.7 |
| C | EH | 2 | 1460 | 35.04 | | 2.13 | 2.38 | 6.3 |
| C | EH | 3 | 812 | 19.49 | | 2.08 | 2.22 | 6.2 |
| C | H | 1 | 660 | 15.84 | | 2.14 | 1.85 | 5.3 |
| C | H | 2 | 635 | 15.24 | | 2.09 | 2.23 | 5.4 |
| C | H | 3 | 480 | 11.52 | | 2.00 | 2.34 | 5.6 |
| C | PW | 1 | 505 | 12.12 | | 2.13 | 2.38 | 6.3 |
| C | PW | 2 | 363 | 8.71 | | 2.14 | 2.23 | 4.8 |
| C | PW | 3 | 840 | 20.16 | | 2.15 | 2.13 | 5.9 |
| C | D0 | 1 | 555 | 13.32 | | 2.10 | 2.28 | 5.7 |
| C | D0 | 2 | 396 | 9.50 | | 2.14 | 2.10 | 5.5 |
| C | D0 | 3 | 539 | 12.94 | | 2.15 | 2.37 | 5.8 |
| C | D7 | 1 | 506 | 12.14 | | 2.13 | 2.16 | 5.4 |
| C | D7 | 2 | 476 | 11.42 | | 2.13 | 2.14 | 4.9 |
| C | D7 | 3 | 396 | 9.50 | | 2.06 | 2.00 | 4.6 |
| C | SC | 1 | 122 | 2.81 | | 2.18 | 1.79 | 4.3 |
| C | SC | 2 | 106 | 2.43 | | 2.21 | 1.96 | 5.2 |
| C | SC | 3 | 282 | 6.77 | | 2.10 | 2.20 | 6.0 |
