# Supplementary material for: The Eruca sativa Genome and Transcriptome: A Targeted Analysis of Sulfur Metabolism and Glucosinolate Biosynthesis Pre and Postharvest
Source: Front Plant Sci. 2020 Oct 27;11:525102. doi: 10.3389/fpls.2020.525102 (PMC7652772; doi:10.3389/fpls.2020.525102)
Supplement: Supplementary Table 3 — qRT-PCR primers and efficiencies. [file Table_3.DOCX]

| **Table S3.** qRT-PCR primers and efficiencies | | | | | | | |
| --- | --- | --- | --- | --- | --- | --- | --- |
| **Gene** | **Oligo** | **Length (bp)** | **tm** | **GC%** | **Sequence** | **Product size (bp)** | **Efficiency** |
| *MYB122-1a* | LEFT | 20 | 62.45 | 60.00 | GGCGAACCCTACCCGACAAA | 163 | 2.13 |
|  | RIGHT | 23 | 60.31 | 43.48 | GTGGACCATTTGTTGCCATGAAT |  |  |
| *MYB51a* | LEFT | 19 | 60.00 | 57.89 | GGCGAACTCTCCCCGAAAA | 164 | 1.93 |
|  | RIGHT | 21 | 62.29 | 52.38 | TGCAGCCCATTTGTTTCCGTG |  |  |
| *BCAT4* | LEFT | 20 | 62.35 | 55.00 | TCGTCTCCGCCGTCAAACAA | 198 | 1.91 |
|  | RIGHT | 20 | 61.55 | 55.00 | ACCCCGCGTTATCCTTGTGA |  |  |
| *SOT16* | LEFT | 21 | 63.06 | 57.14 | CCCAACACAACGGACACTGGT | 187 | 1.77 |
|  | RIGHT | 20 | 62.74 | 60.00 | AGAGGGTTCGTTGCGTCGTC |  |  |
| *CYP83B1* | LEFT | 23 | 59.73 | 43.48 | TTTGGATATTGTTGTACCCGGGA | 150 | 2.03 |
|  | RIGHT | 20 | 59.12 | 55.00 | TCTTCTTCCGAGACGTGTCC |  |  |
| *SUR1* | LEFT | 22 | 61.94 | 50.00 | TCATTCAGGCTGCACTTCCTCA | 105 | 1.90 |
|  | RIGHT | 23 | 61.17 | 47.83 | GCCTATCACACACCAAATCGACA |  |  |
| *TGG1b* | LEFT | 20 | 61.91 | 55.00 | AGCTTCTTCATGGCCTCGCT | 190 | 1.93 |
|  | RIGHT | 22 | 63.20 | 59.09 | CCCCTCCCTCCTTCAATCTGGT |  |  |
| *TGG1j* | LEFT | 21 | 61.51 | 52.38 | TGGTACATGGAGCCGCTTACA | 159 | 2.08 |
|  | RIGHT | 21 | 61.76 | 57.14 | TGACTGGGCGTACTGAGTGAC |  |  |
| *UGT74B1a* | LEFT | 20 | 62.99 | 65.00 | CAGCATCGACGCCTACTCCG | 247 | 1.71 |
|  | RIGHT | 20 | 61.26 | 60.00 | GGGAACAGCGGGGAGAGTAA |  |  |
| *TGG1d* | LEFT | 21 | 62.26 | 52.38 | TCAGAAGACCGTTGCCAAGCT | 104 | 1.84 |
|  | RIGHT | 22 | 61.55 | 50.00 | ACGTTGACACCCTTCTCCTTGA |  |  |
| *ACT11* | LEFT | 20 | 62.99 | 60.00 | TTCACCACCACAGCAGAGCG | 165 | 2.19 |
|  | RIGHT | 20 | 62.59 | 65.00 | CCTCTCCCCTCCGATGGTGA |  |  |
