# Supplementary material for: The Eruca sativa Genome and Transcriptome: A Targeted Analysis of Sulfur Metabolism and Glucosinolate Biosynthesis Pre and Postharvest
Source: Front Plant Sci. 2020 Oct 27;11:525102. doi: 10.3389/fpls.2020.525102 (PMC7652772; doi:10.3389/fpls.2020.525102)
Supplement: Supplementary Table 4 — Numbers of genes with homology or functional assignment. [file Table_4.DOCX]

| **Table S4.** Numbers of genes with homology or functional assignment | | | |
| --- | --- | --- | --- |
| **Database** | | **Number of annotated genes** | **Gene annotation %** |
| NR | | 44,630 | 98.2 |
| Swiss-Prot | | 35,309 | 77.7 |
| KEGG | | 32,589 | 71.7 |
| InterPro | All | 37,067 | 81.6 |
|  | Pfam | 34,541 | 76 |
|  | GO | 25,820 | 56.8 |
| Annotated | | 44,655 | 98.3 |
| **Total** | | **45,438** |  |
